# Comparison of the ACASI Mode to Other Survey Modes in Sexual Behavior Surveys in Asia and Sub-Saharan Africa: Systematic Literature Review

**DOI:** 10.2196/37356

**Published:** 2022-05-31

**Authors:** Nang Nge Nge Phoo, Roanna Lobo, Daniel Vujcich, Alison Reid

**Affiliations:** 1 School of Population Health Curtin University Perth Australia

**Keywords:** ACASI, survey mode, sexual behaviors, HIV, STI, hepatitis, blood-borne virus, Asia, sub-Saharan Africa, review

## Abstract

**Background:**

Reliable data about sexual behaviors is fundamental in the prevention and control of HIV, hepatitis, and other sexually transmitted infections. Generally, sexual behaviors are regarded as a sociocultural taboo in Africa and Asia, and this results in biased sexual behavior survey data due to social desirability. Various modes of survey delivery, including audio computer-assisted self-interviews (ACASIs), have been investigated to improve data quality.

**Objective:**

This study aimed to review studies that compared the ACASI mode to other survey modes in sexual behavior surveys in Asia and sub-Saharan Africa to ascertain the impact of survey mode on responses to sexual behavior questions.

**Methods:**

A systematic literature review was conducted according to the Joanna Briggs Institute Manual for Evidence Synthesis. The review protocol was registered at PROSPERO (International Prospective Register of Systematic Reviews). Six databases were searched.

**Results:**

A total of 21 papers were included. The face-to-face interview (FTFI) mode was the survey mode most frequently compared to the ACASI mode. Among the most commonly reported outcome variable groups, ACASI participants were more likely to report sexual behaviors, such as “forced sex,” “multiple partners,” “transactional sex,” and “ever had sex,” as compared to FTFI participants. In addition to the survey mode effect, other factors were found to have had an impact on data quality, for example, participant characteristics, social norms, study design, and data collection setting.

**Conclusions:**

Use of ACASIs for administering sexual behavior surveys among populations in Asia and sub-Saharan Africa demonstrated higher reports for some sexual behaviors than the use of FTFIs. More studies that compare the ACASI mode to other survey modes would improve our understanding of the usefulness of ACASIs in sexual behavior surveys in these regions.

## Introduction

While the annual number of new HIV cases decreased globally over the last decade from 2.1 million in 2010 to 1.5 million in 2020, there remained disparities across regions [1]. The HIV infection rate per 1000 uninfected population members was highest in the World Health Organization (WHO) African Region in 2020 (ie, 0.82 in Africa compared to 0.18 and lower in other regions) [1]. Similarly, in 2020, HIV-related deaths were higher in the WHO African Region (n=460,000) and in Southeast Asia (n=82,000) compared to other regions (n≤45,000) [1]. Furthermore, the prevalence rates of hepatitis B infection and other sexually transmitted infections (STIs) were highest in the WHO African Region compared to all other regions [2-4].

Reliable data about sexual behaviors is fundamental in the prevention and control of HIV, hepatitis, and other STIs. Such data can help predict the transmission of STIs and inform strategies to prevent their transmission [5,6]. Development of intervention strategies requires exploration of the presence or frequency of each behavior and the risk and protective factors associated with each behavior [5,6]. Despite frequent research around sexual behaviors in Africa, this information was scarce for Asia [7].

Information on sexual behaviors is largely collected through self-reported knowledge, attitude, and practice surveys, for example, sexual behavior surveys in Thailand, Kenya, the United States, European countries, and Middle Eastern countries [8-11]. However, it is well recognized that the quality of self-reported data can be undermined in research about socially stigmatized behaviors [12-15]. Generally, “sexual behaviors” as a topic is a sensitive one and is regarded as taboo in Africa and Asia [16-19]. Consequently, attempts have been made to understand whether certain aspects of survey design can improve the quality of self-reported sexual behavior data. Previous research noted that the factors that impact on responses in sexual behavior surveys include the surveyed population, social context, respondent variables, an individual’s choice, study design, data collection tools, and survey modes [20-22]. For instance, regarding survey modes, the involvement of interviewers can lead to a lack of privacy and increased social desirability bias, whereas self-administered surveys require minimum levels of literacy [12,15].

An audio computer-assisted self-interview (ACASI) is an electronic survey in which respondents listen to prerecorded survey questions, while reading the survey questions on desktops, laptops, or touch-screen devices, and enter their responses using a mouse, keyboard, color- or number-coded keypad, or touch screen [23]. Many studies have tested the use of ACASIs in capturing sensitive information with the hypotheses that noninvolvement of interviewers would enhance privacy and that the speech function would help respondents overcome any literacy issues, thus decreasing item nonresponse rates and increasing the prevalence of self-reported sexual experiences [24-27]. There have been a few reviews of studies comparing responses obtained from different survey modes, including ACASI and other computerized administration modes [24-27], with responses obtained from more traditional survey modes (eg, face-to-face interviews [FTFIs]). A review of 26 studies examining sexual behaviors of adolescents and adults in developing countries, in which 18 studies compared responses from ACASIs with those from other survey modes, found a higher rate of reporting of sensitive behaviors with computer-assisted interviews than with the other modes [24]. One of the papers included in that review reported that women were more likely to report anal sex in ACASIs (33%) than in FTFIs (24%; *P*<.01) [24,28]. A similar conclusion was reported by a meta-analysis of 48 studies, mostly from the United States and Europe [26], which compared the prevalence of self-reported sexual behaviors reported in computerized self-administration surveys with the prevalence in paper-and-pencil self-administration surveys regarding sensitive behaviors. A meta-analysis of the results from 15 survey studies about sexual behaviors and intravenous drug use compared reporting rates between face-to-face and non–face-to-face survey methods; in contrast to the previous meta-analysis, this one observed that the ACASI mode was not always associated with higher reporting of such behaviors, except for the studies conducted in Asia, urban areas, and among participants with secondary education and higher [25]. However, more than 60% of the studies assessed in these reviews were conducted in areas other than Africa or Asia, and ACASI was not the main survey mode of interest [24-26]. Therefore, there is a gap in knowledge about the usefulness of using the ACASI mode compared with other survey modes in sexual behavior surveys conducted in Africa and Asia.

Given the high incidence of HIV, hepatitis, and other STIs in the WHO African Region and in Asia, as well as scarce sexual behavior information in Asia, there is a need to identify a survey mode that best collects sensitive information around sexual behaviors for these populations. The aim of this study was to identify and review the studies conducted in Asia and sub-Saharan Africa that compared the ACASI mode to other survey modes in sexual behavior surveys in order to assess the impact of different survey modes on reporting of sexual behaviors.

## Methods

### Overview

A systematic literature review was conducted according to the Joanna Briggs Institute (JBI) Manual for Evidence Synthesis [29]. The review protocol was registered at PROSPERO (International Prospective Register of Systematic Reviews; registration No. CRD42020197237). A systematic search was conducted in six databases: Embase, MEDLINE, ProQuest Public Health Database, PsycInfo, Web of Science, and Global Health. The search commenced in June 2020 and was updated in August 2021. The search terms used were as follows: “ACASI,” “audio computer,” “survey mode,” “HIV,” “AIDS,” “sex,” “STI,” “STD” (sexually transmitted disease), “syphilis,” “chlamydia,” “gonorrh*,” “hepatitis,” “blood-born*,” “BBV” (blood-borne virus), and the names of the countries in Asia and sub-Saharan Africa. The search strategies used in the MEDLINE database are included in Figure S1 and Table S1 in Multimedia Appendix 1.

### Inclusion and Exclusion Criteria

#### Population

The eligible population included adults and adolescents aged 15 years and older living in 36 countries in Southeast Asia, Northeast Asia, South Asia, and Central Asia, and in 48 countries in sub-Saharan Africa as listed by the World Bank in 2020 [30]. The population was limited to adults and mature young adults because this review was conducted as formative research for the original research which would have been carried out among adult populations.

#### Intervention

The intervention of interest was the use of ACASI in surveys about sexual behaviors and knowledge, attitudes, and practices around HIV, other STIs, and BBVs. The studies that focused on antiretroviral therapy and contraception use or adherence were not selected.

#### Comparators

The comparators to the ACASI mode were other survey modes with or without biological marker tests. When the ACASI mode was compared to biological markers only, the studies were excluded.

#### Outcomes

The outcome of interest was the difference in odds or prevalence of self-reported sexual behaviors between the ACASI mode and other survey modes. Moreover, the comparisons of discordance between the ACASI mode and biological marker results and that between other survey modes and biological marker results were also examined. Studies were also excluded when the outcome of interest was only the comparison in item response or refusal rates and user experience between the ACASI mode and other survey modes.

#### Resource Type, Publication Language, and Publication Period

Only peer-reviewed articles of primary studies published in English from 2000 to July 2021 were included.

#### Screening

The titles and abstracts of search results were screened against inclusion and exclusion criteria. The full text of papers with relevant titles and abstracts were retrieved and checked. The papers with relevant full text were included. The bibliographies of the included papers and the papers that cited the included papers were screened against the eligibility criteria. The search and screening results are presented in a 2020 PRISMA (Preferred Reporting Items for Systematic Reviews and Meta-Analyses) flow diagram (see Results section) [31].

### Generic Study Characteristics and Delivery of the ACASI Mode

Data extraction was performed according to the JBI data extraction form for prevalence studies [29]. This included citation details, the generic study details, and description of main results. In addition, details relating to the focus of this review were extracted, including modes of survey administration, how the ACASI survey was delivered, and number of people or odds of reporting the presence of each sexual behavior by each survey mode. The generic study characteristics were summarized using tables, and a narrative summary was developed for the study characteristics and the delivery of the ACASI mode (see Results section).

### Methodological Quality Appraisal

The methodological quality of included studies was appraised using the JBI critical appraisal instrument for systematic reviews of prevalence and incidence [32]. There were nine appraisal criteria, which assessed sampling bias, adequacy of sample size, generalizability of study findings, validity of study methods, measurement bias, and the use of appropriate statistical analysis methods. Each criterion was appraised as “yes,” “‘no,” “‘not clear,” or “‘not applicable.” To summarize for reporting purposes, each criterion that was scored as “yes” was assigned a value of 1 and those scored as “no,” “not clear,” or “not applicable” were assigned a value of 0.

### Comparison of Survey Modes

To compare sexual behavior outcomes, similar outcome variables were grouped; for example, “buy sex,” “‘sell sex,” “paid for sex,” and “received cash for sex” were grouped together and named “transactional sex.” For each group, the effect sizes that reported on the comparison between the ACASI mode and other survey modes were tabled. Since only a few effect sizes were reported for the comparison between the ACASI mode and the survey modes other than FTFI, only the comparisons between ACASI and FTFI modes were included in the analysis. The effect sizes were grouped based on statistical reports, such as odds ratio (OR), prevalence, predicted percentage, count, mean, and median. When such information was missing, the corresponding authors were contacted for the required information. Because the OR and prevalence values were the most commonly reported, these two types of effect sizes were the focus for this analysis.

When the reviewed papers reported ORs with CIs for the comparison of reporting sexual behaviors between ACASI and FTFI modes, the results were included in the analyses. When the percentages of respondents reporting a sexual behavior or experience and denominators were reported, crude ORs were calculated, with FTFI as the comparative mode. The crude ORs were calculated as the odds of reporting a sexual behavior in the ACASI group divided by the odds of reporting a sexual behavior in the FTFI group. Crude ORs that were greater than 1 were interpreted as follows: the reporting of sexual behaviors was higher with the ACASI mode than with the FTFI mode. For comparisons including biological markers, crude ORs greater than 1 were interpreted as follows: the odds of the discordance between ACASI self-reports and biological marker results were higher than the discordance between FTFI self-reports and biological marker results. For each outcome variable group with eight or more ORs with CIs available, a forest plot was used to visualize the effect sizes reported for each variable group. In addition, the comparison of sexual behavior reports between the ACASI or FTFI mode and biological marker results was charted. No subgroup analysis or meta-analysis was performed due to the heterogeneity of outcome measures and study populations. The heterogeneity for each variable group ranged from 23.8% to 95.7%.

## Results

### Overview

**Figure 1:**
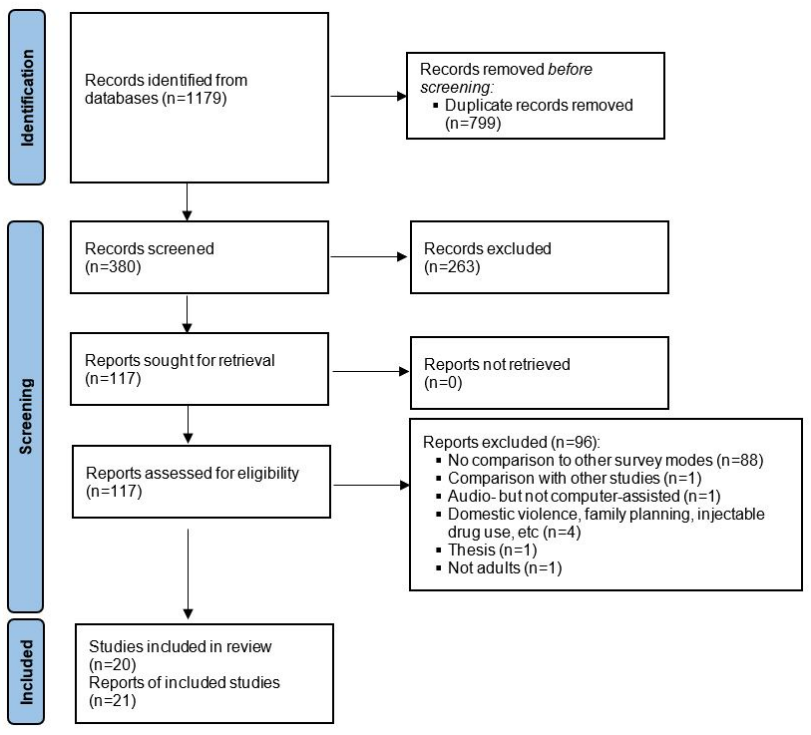
2020 PRISMA flow diagram of the search and screening results of the systematic literature review. The review compared the reporting of sexual behaviors in surveys between those using the ACASI mode and other modes in Asia and sub-Saharan Africa. ACASI: audio computer-assisted self-interview; PRISMA: Preferred Reporting Items for Systematic Reviews and Meta-Analyses.

The search of six databases identified 1179 papers, of which 799 were duplicates (Figure 1). The titles and abstracts of 380 papers were screened, and 263 were removed for reasons such as not testing the ACASI mode, the survey topic not being “sexual behaviors,” the study not originating from Asia or Africa, or participants being younger than 15 years of age. The full texts of 117 papers were then read and, following this, a further 96 papers were excluded; this left 21 papers. The screening of 650 references cited by the 21 papers, as well as 1049 papers that cited the 21 papers, did not result in the inclusion of any more relevant papers. Among the 21 papers, 2 presented different analysis results of the same study [33,34]. Therefore, a total of 21 papers from 20 different studies were included in this review. The search and screening results are summarized in Figure 1.

### Generic Study Characteristics

The generic study characteristics are summarized in Table 1 [33-53]. Among the 20 studies, 65% (n=13) were experimental, quasi-experimental, or randomized trials, while the remaining studies (n=7, 35%) were cross-sectional or longitudinal surveys. More than half of the studies (n=12, 60%) were from sub-Saharan Africa, and the remaining studies (n=8, 40%) were from Asia. The number of participants ranged from 180 to 6530, and all participants were aged 15 years or older. All of the included studies compared the ACASI mode to at least one other survey mode (Table 2). More than half of the studies (n=11, 55%) compared the ACASI mode to only one other survey mode. The FTFI mode was the most compared mode in the reviewed studies (n=17, 85%). In half of the studies (n=10, 50%), the participants used more than one survey mode.

**Table 1:** Generic study characteristics and quality appraisal from the papers included in the systematic literature review comparing the ACASI^a^ mode to other survey modes in sexual behavior surveys in Asia and sub-Saharan Africa.

Author, year	Country	Participants, n	Age (years), range	Females, %	Quality appraisal score^b^
Mensch, 2003 [33]	Kenya	6530	15-21	—^c^	6
Hewett, 2004 [34]	Kenya	709	15-21	100	—
Potdar, 2005 [35]	India	1500	18-22	0	8
Griensven, 2006 [36]	Thailand	1283	15-21	50.1	9
Le, 2006 [37]	Vietnam	2394	15-24	54.3	7
NIMH^d^, 2007 [38]	China, India, Peru, Russia and Zimbabwe	445	16-40	48.9	3
Minnis, 2007 [39]	Zimbabwe	655	18-35	100	5
Li, 2007 [40]	China	199	—	45.2	6
Edwards, 2008 [41]	Russia	180	≥18	25	5
Jaya, 2008 [42]	India	1058	15-19	44.9	9
Mensch, 2008 [43]	Malawi	447	15-21	100	7
Minnis, 2009 [44]	Zimbabwe	910	18-49	100	4
van der Elst, 2009 [45]	Kenya	398	—	34.9	3
Mensch, 2011 [46]	South Africa	849	18-40	100	8
Langhaug, 2011 [47]	Zimbabwe	1495	15-23	44.7	8
Le, 2012 [48]	Vietnam	4049	15-49	54.9	7
Gorbach, 2013 [49]	Malawi	585	18-53	100	4
Kelly, 2013 [50]	Malawi	311	16-18	37.3	5
Adebajo, 2014 [51]	Nigeria	1040	≥18	0	3
Kelly, 2014 [52]	Uganda	1020	18-24	100	7
Desmond, 2018 [53]	Malawi	300	16-49	48	6

^a^ACASI: audio computer-assisted self-interview.

^b^The quality appraisal score ranges from 0 to 9.

^c^Not reported.

^d^NIMH: National Institute of Mental Health.

**Table 2:** Survey modes and outcomes of studies from the papers included in the systematic literature review comparing the ACASI mode to other survey modes in sexual behavior surveys in Asia and sub-Saharan Africa.

Author, year	Modes, n^a^	Modes of survey administration					Modes per participant, n		Outcomes^b^	
		ACASI^c^	FTFI^d^	SAQ^e^	Other modes			Difference		Validation
Mensch, 2003 [33]	3	✓^f^	✓	✓		1		✓		
Hewett, 2004 [34]	2	✓	✓			1		✓		
Potdar, 2005 [35]	3	✓	✓	✓		1		✓		
Griensven, 2006 [36]	4	✓	✓	✓	PASI^g^	1		✓		
Le, 2006 [37]	3	✓	✓	✓		1		✓		
NIMH^h^, 2007 [38]	2	✓			CAPI^i^	2 (crossover)		✓		
Minnis, 2007 [39]	2	✓	✓			2 (crossover)		✓		
Li, 2007 [40]	2	✓			CAPI	2 (crossover)		✓		
Edwards, 2008 [41]	2	✓	✓			2		✓		
Jaya, 2008 [42]	3	✓	✓		Interactive^j^	2		✓		
Mensch, 2008 [43]	2	✓	✓			1		✓		
Minnis, 2009 [44]	2	✓	✓		PSA^k^	1		✓		✓
van der Elst, 2009 [45]	2	✓	✓			2		✓		
Mensch, 2011 [46]	2	✓	✓		RSID^l^	1		✓		✓
Langhaug, 2011 [47]	4	✓		✓ (with audio)	ICVI^m^	2		✓		
Le, 2012 [48]	3	✓	✓	✓		1		✓		
Gorbach, 2013 [49]	2	✓	✓			2		✓		
Kelly, 2013 [50]	2	✓	✓			2		✓		
Adebajo, 2014 [51]	2	✓	✓			1		✓		
Kelly, 2014 [52]	3	✓	✓		CAPI and RSID	1		✓		✓
Desmond, 2018 [53]	3	✓	✓ (with visual recall aids)		Daily pictorial diary	3		✓		

^a^Number of modes of survey administration.

^b^“Difference” represents the differences in reporting of sexual behaviors across survey modes; “validation” represents validation using biological markers.

^c^ACASI: audio computer-assisted self-interview.

^d^FTFI: face-to-face interview.

^e^SAQ: self-administered questionnaire.

^f^A checkmark indicates that the respective study included the indicated mode or outcome.

^g^PASI: palmtop-assisted self-interview.

^h^NIMH: National Institute of Mental Health.

^i^CAPI: computer-assisted personal interview.

^j^Interactive interview using audio-visual aids and confidential voting.

^k^PSA: prostate-specific antigen (biological marker).

^l^RSID: rapid stain identification of human semen (biological marker).

^m^ICVI: informal confidential voting interview.

### Delivery of the ACASI Mode

Laptops were the most commonly used devices to deliver the ACASI mode (n=9, 45%), while the remaining studies used desktops, handheld computers, and palmtops. In total, 6 (30%) studies used touch-screen devices, and all of these studies were published after 2005. Color- or number-coded keypads were used in 6 (30%) studies. The use of headphones was reported in 12 (60%) studies. The narration was only in a female voice for 2 (10%) studies, in both genders for 4 (20%) studies, and unreported in the other 14 (70%) studies.

### Methodological Quality Appraisal

The sample frame was well defined in the majority of studies (n=17, 85%). Random sampling was used in 15 (75%) studies. The sample size calculation was reported in 8 (40%) studies, and it was adequate in 7 (35%) studies. The study participants and settings were described in detail in the majority of studies (n=18, 90%). The response rate for each survey mode arm was reported in only 7 (35%) studies. The validity of the questionnaire was reported in 9 (45%) studies. The surveys were reliably delivered by trained data collectors in most studies (n=14, 70%). The statistical analysis was appropriate in all the studies. The response rate was 74% and higher in more than half of the studies reviewed (n=13, 65%). On a scale of 0 to 9, the total quality appraisal score for the reviewed studies was 7 to 9 (n=9, 45%), 4 to 6 (n=8, 40%), and 3 (n=3, 15%). The quality appraisal scores are listed in Table 1 [33-53].

### Comparison of Survey Modes

**Figure 2:**
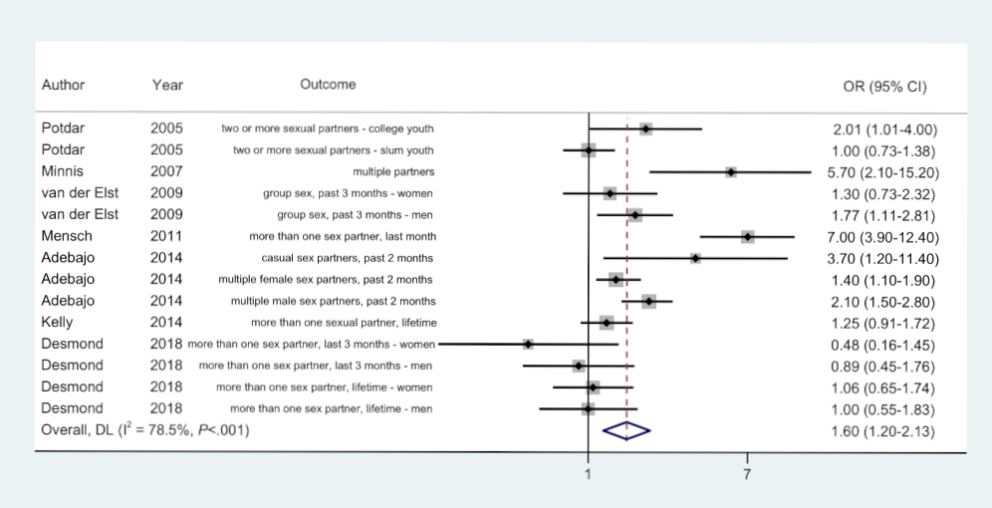
Random-effects model. Odds ratios (ORs) of reporting "multiple sexual partners" in the ACASI mode as compared to the FTFI mode. ACASI: audio computer-assisted self-interview; DL: DerSimonian-Laird effect size variance estimates; FTFI: face-to-face interview.

**Figure 3:**
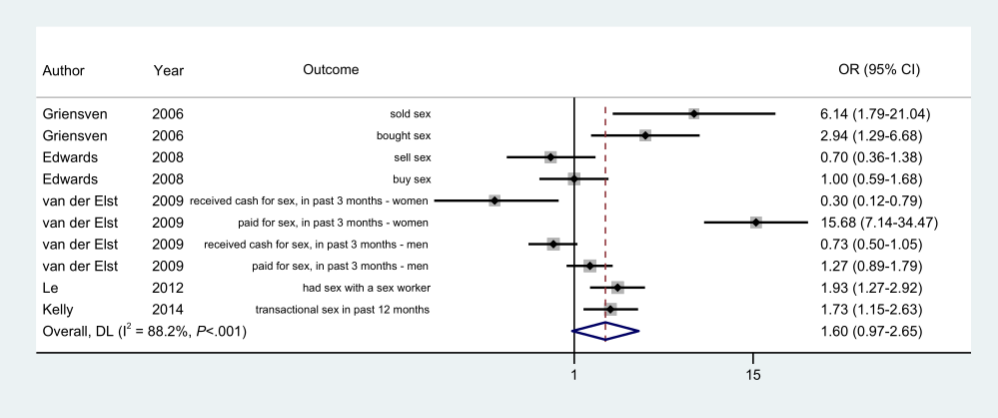
Random-effects model. Odds ratios (ORs) of reporting "transactional sex" in the ACASI mode as compared to the FTFI mode. ACASI: audio computer-assisted self-interview; DL: DerSimonian-Laird effect size variance estimates; FTFI: face-to-face interview.

**Figure 4:**
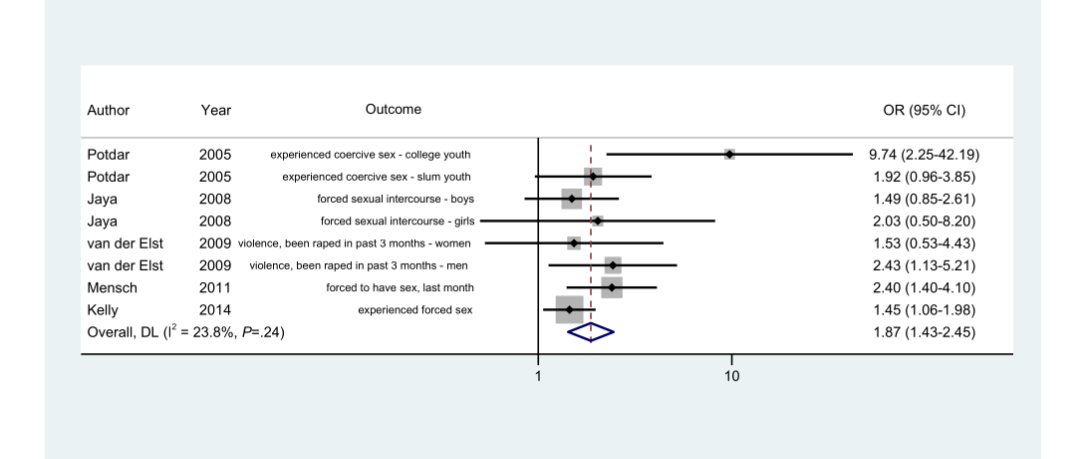
Random-effects model. Odds ratios (ORs) of reporting "forced sex" in the ACASI mode as compared to the FTFI mode. ACASI: audio computer-assisted self-interview; DL: DerSimonian-Laird effect size variance estimates; FTFI: face-to-face interview.

**Figure 5:**
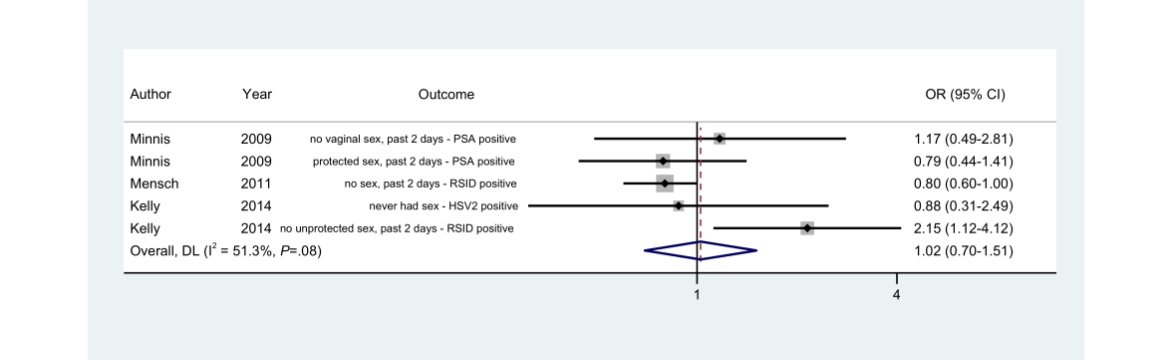
Random-effects model. Odds ratios (ORs) of the "discordance between self-reports and biological marker results" in the ACASI mode as compared to the FTFI mode. ACASI: audio computer-assisted self-interview; DL: DerSimonian-Laird effect size variance estimates; FTFI: face-to-face interview; HSV2: herpes simplex virus type 2; PSA: prostate-specific antigen; RSID: rapid stain identification of human semen.

The grouping of similar sexual behavior outcome variables resulted in 23 groups (Table S2 in Multimedia Appendix 1). Among the comparisons between the ACASI mode and other survey modes, the majority were between ACASI and FTFI modes (n=17, 85%). The crude ORs with CIs were available for eight or more comparisons for seven groups of outcome variables, namely, condom use, multiple sexual partners, ever had sex, ever had a partner, transactional sex, anal sex, and forced sex. There were five comparisons between sexual behavior self-reports and biological markers. For these seven groups and the comparison with biological marker results, the effect sizes were visually presented using forest plots (Figures 2-5 and Figures S2-S5 in Multimedia Appendix 1). For the remaining 16 groups of outcome variables, due to the small number of similar outcome measures, the results are summarized in Table S2 in Multimedia Appendix 1, but are not plotted in a figure or included in the narrative report.

### Multiple Sexual Partners

A total of 7 out of 20 (35%) studies reported 14 effect sizes for comparing the odds of reporting multiple sexual partners between ACASI and FTFI modes. Participants who used the ACASI mode were more likely to report having multiple sexual partners than those who used the FTFI mode in 10 out of 14 (71%) comparisons, were just as likely in 2 (14%) comparisons, and were less likely in 2 (14%) comparisons (Figure 2). The time frames differed, as follows: the last 1 to 3 months, lifetime, and nonspecified time frame. The wording for “multiple” varied, such as “more than one,” “two or more,” “multiple,” “casual partners,” and “group sex.” The prevalence of having multiple sexual partners was significantly higher among ACASI participants than FTFI participants in the study that recruited men who had sex with men and men who injected drugs [51]. Similarly, the ACASI participants were 6.4 times (95% CI 3.6-11.2) more likely to report having more than one partner than FTFI participants, and the effect size increased to 7 (95% CI 3.9-12.4) when adjusted for age, marital status, education, ethnicity, and study site (*P*<.01) [46]. The odds of reporting multiple sexual partners were higher for ACASI participants than FTFI participants in the Zimbabwean study, which reported ORs for the second visit; however, this result might have been affected by the longitudinal crossover design [39]. The results differed across subgroups within the same study. The behavior of having multiple partners was more frequently reported by participants using the ACASI mode than the FTFI mode among college-attending youth in the study in India, whereas there was no such difference for slum youths [35]. Likewise, the difference in the self-reported prevalence of multiple partners between the two modes (ie, ACASI vs FTFI) was significant among male participants but not significant among female participants of the study in Kenya [45].

### Transactional Sex

The outcome measures related to “transactional sex” were evaluated using ACASI and FTFI modes in 5 out of 20 (25%) studies, and 10 effect sizes were reported (Figure 3). The prevalence of transactional sex was higher in the ACASI arm for 6 (60%) comparisons, it was higher in the FTFI arm for 3 (30%) comparisons, and there was no difference for 1 (10%) comparison. The ACASI participants were more likely to report transactional sex than FTFI participants for 6 (60%) effect sizes, of which 5 (83%) were statistically significant.

### Forced Sex

The odds of reporting forced sex were higher among ACASI participants than FTFI participants for all 8 results reported by 5 (25%) studies (Figure 4). The wording of outcomes varied, as follows: “forced,” “coercive,” “violence,” and “rape.” The higher reports of forced sex among ACASI participants than FTFI participants were significant in the South African and Ugandan studies, in which all participants were women, but both male and female interviewers were involved in the FTFI mode [46,52]. The difference between modes was not statistically significant in the Indian study among unmarried girls and boys aged 15 to 19 years [42]. In the study among sex workers, despite the overlapping CI for women participants, the difference in prevalence was statistically significant (ACASI: 6.6% vs FTFI: 4.4%; n=139; *P*=.40) [45]. The reports of forced sex were statistically significantly higher when the surveys were administered with ACASIs than with FTFIs among college-attending youth but not slum-dwelling youth in India [35].

### Condom Use

The odds of reporting condom use were compared between ACASI and FTFI participants in 8 out of 20 (40%) studies, and there were 16 effect sizes. The odds of reporting condom use were lower among ACASI participants than FTFI participants for 7 out of 16 (44%) reports, higher for 7 (44%), and the same for 2 (13%; Figure S2 in Multimedia Appendix 1). Condom use outcomes had different time frames, such as last sexual encounter, last week, last 3 or 6 months, and nonspecified time frame. There were differences across the studies, such as the study populations, the number of survey modes applied to each participant, and study objectives. All the participants were female in 3 of the 8 (38%) studies [39,49,52]. Populations that were considered high risk were recruited in 2 (25%) studies; these were female and male sex workers in Kenya and patients admitted to the substance abuse treatment hospitals in Russia [41,45]. The participants of the studies in Zimbabwe, Russia, South Africa, and Malawi were administered surveys via both ACASI and FTFI modes [39,41,49,53]. In the study in South Africa, the participants were administered surveys via only one of the two survey modes; however, they had to take the same survey on four occasions [46]. The authors of two microbicide trials noted that participants were likely to overreport condom use due to the adherence counseling they had received [49], and the privacy provided via the ACASI mode alone did not assure disclosure of nonadherence to condom use [46].

### Ever Had Sex

The variables related to “ever had sex” were compared between the ACASI and FTFI modes in 5 out of 20 (25%) studies, and 13 effect sizes were reported (Figure S3 in Multimedia Appendix 1). The participants in the ACASI arm reported having had sex more frequently than those in the FTFI arm for 8 (62%) effect sizes, of which only 1 (13%) was statistically significant [35]. Out of 5 studies, 2 (40%) disaggregated the findings by sex, and the higher frequency of reporting ever having had sex among ACASI participants compared to FTFI participants was more prominent among females than males, although the mode difference was not statistically significant [42,50].

### Ever Had a Partner

For the outcome variable group “ever had a partner,” out of 20 studies, 1 (5%) study in Malawi and 2 (10%) studies in India reported 12 effect sizes (Figure S4 in Multimedia Appendix 1). The odds of reporting ever having had a partner was higher among FTFI participants for 6 (50%) comparisons, higher among ACASI participants for 5 (42%) comparisons, and there was no difference between the two modes for 1 (8%) comparison. In a study in India, the young men recruited from colleges and slums reported same-sex relationships more frequently via the ACASI mode than the FTFI mode, and the mode difference was significant for college youths (OR 7.84, 95% CI 1.78-34.59; *P=*.007) [35].

### Anal Sex

The survey mode comparison for the outcomes related to “anal sex” was performed in 5 out of 20 (25%) studies, which reported 9 effect sizes (Figure S5 in Multimedia Appendix 1). The reporting of anal sex was more common among ACASI participants than FTFI participants for 4 (44%) effect sizes, and all were statistically significant.

### Comparison With Biological Markers

Self-reports of sexual behaviors via the ACASI and FTFI modes were compared to the biological marker results in 3 out of 20 (15%) studies, and 5 outcomes were reported (Figure 5). The biological marker tests used in these studies included herpes simplex virus type 2, prostate-specific antigen, and rapid stain identification of human semen (RSID). The subjective reports of no unprotected sex in the past 2 days and objective findings of the presence of sex were more significantly discordant among ACASI participants than FTFI participants in the study by Kelly et al [52]. The mode difference was marginally significant, with FTFI participants reporting more discordantly with biological marker results than ACASI participants in the study by Mensch et al [46], since ACASI participants were 0.8 times more likely than FTFI participants to report no sex in the past 2 days despite positive RSID results (95% CI 0.6-1.0; *P*=.10).

## Discussion

### Principal Findings

A total of 21 papers that reported the results from 20 different studies comparing the reporting of sensitive questions via the ACASI mode and other survey modes were reviewed in this study. The methodological quality of the reviewed papers was moderate to good. The FTFI mode was the most commonly compared mode to ACASI. Among the seven most commonly examined groups of outcome variables, the participants in the ACASI arm were more likely to report having had forced sex, multiple partners, and transactional sex, as well as ever having had sex than the FTFI arm. The odds of reporting were either higher or lower among ACASI participants compared to FTFI participants for condom use, having a partner, and anal sex. When validated with biological markers for five comparisons, there was no significant difference in three, discordance was higher with ACASI in one, and discordance was higher with FTFI in another.

### Comparison With Prior Work

The odds of reporting sexual behaviors, such as forced sex, multiple partners, transactional sex, and having had sex, were higher when the surveys were administered with the ACASI mode than with the FTFI mode. This finding was similar to other reviews that showed higher reporting of sexual behaviors with computer-assisted survey administration [24,27]. A meta-analysis of 48 studies was conducted mostly in the United States and Europe and compared reporting rates of socially undesirable behaviors when using interviewer-delivered and computerized survey modes; the analysis concluded that self-disclosure was higher in the latter, and such differences were more pronounced for highly sensitive behaviors [26]. A meta-analysis of 15 surveys compared reporting rates of HIV risk behaviors in low- and middle-income countries when using FTFIs compared to non-FTFI methods; the analysis reported consistently higher reporting of “forced sex” with non-FTFI methods [25].

Despite higher reporting of sexual behaviors with the ACASI mode compared to the FTFI mode for a few outcome variables, the validation against biological marker results suggested that computerized delivery alone was not always associated with reliable self-reports. This inference was supported by a review of the use of subjective and objective methods in sexual behavior assessment, which concluded that objective assessment was associated with more reliable results [54]. Similarly, a review of survey modes to administer HIV and other STI risk behavior surveys concluded that self-reported prevalence of such risk behaviors was not always high with non-FTFI modes, such as ACASIs, assisted self-administered questionnaires, informal confidential voting interviews, palmtop-assisted self-interviews, polling booth surveys, and tape-recorded interviews [25].

The reviewed studies noted that the self-reported prevalence of forced sex, multiple partners, transactional sex, and having had sex were higher with the ACASI mode than with the FTFI mode; however, the suitability of the use of ACASIs and the self-reports when using the ACASI mode were based on many factors, such as consequences of not involving interviewers, participants’ sociodemographic factors, literacy, familiarity with the use of computers or similar devices, participants’ conflicts of interest regarding participation in the study, social norms, data collection setting, and study design. These findings were consistent with literature reviews evaluating the impact of inquiry mode on sexual behavior, which reported that many factors, including survey modes, could increase or decrease the reporting of sexual behaviors, and those factors were interactive [22,55]. A few participants highlighted not being able to seek advice from interviewers with the ACASI mode [47]. Similarly, Mensch et al [46] warned that unless a logic check was programmed with the ACASI mode, there would be internally inconsistent responses, which could have been corrected by interviewers via the FTFI mode; for example, when a question about the number of sexual partners was answered with any number greater than zero following a question about ever having had sex being answered as “no,” the logic check prompt feature in the ACASI mode would point out the inconsistency and ask the participants to re-enter their response for the previous question.

The heterogeneity of participant characteristics, culture, and social norms within and across the studies reviewed could have confounded the differences across survey modes. Literacy and familiarity with the use of computers and similar devices were participant prerequisites for the ACASI mode. The studies in China, India, and Vietnam concluded that the participants who were familiar with the use of computers were more comfortable taking the ACASI survey [35,40,42,48]. However, in a study in Kenya in 2000, the participants saw the computers as superstitious, especially when it appeared that the computer was talking to them [33]. van der Elst et al [45] remarked that in a population with limited access to health care, study participants could exaggerate risky behaviors such that they would receive benefits in health care through participation in research. The authors of two reviewed studies suggested that sexual behavior reports might have been affected by the cultural conservatism in India and the Confucian culture in Vietnam [35,37].

The data collection setting could also have affected data quality. When data collection took place in a room with around six or seven participants at the same time, the participants felt a lack of privacy, using either ACASI or FTFI modes [35]. In a Russian study, the authors noted that a common problem of data leakage in Russia (eg, selling personal information on the street) could have impaired the study participants’ trust in the ACASI mode [41]. In studies with a crossover design, the experience in the first mode could have affected the responses in the subsequent modes [38-40,49]. When participants had to take surveys via both modes on the same day and assumed that their responses would be compared, they might have attempted to keep their responses consistent [41]. Desmond et al [53] suggested that any retrospective survey mode might have an issue with memory recall.

### Limitations

There are a few limitations in this study. Since only a limited number of studies from each country or region were identified, the results might not be generalizable to similar populations in corresponding countries or regions. Due to heterogeneity of outcome measures and comparison of the ACASI mode to different survey modes, no meta-analysis or subgroup analysis was performed and, hence, no definitive conclusion was reached. Since this review included only studies published in English, studies published in other languages were missed.

### Conclusions

The self-reported prevalence rates of a few sensitive sexual behaviors were higher with the ACASI mode than with the FTFI mode, for example, forced sex, multiple sexual partners, transactional sex, and ever had sex. However, a mix of higher and lower reporting of other sexual behaviors in the ACASI mode compared to the FTFI mode, as well as discordance between self-reports with the ACASI mode and biological marker results, suggested that an absence of interviewers in the ACASI mode alone did not lower social desirability bias or improve data quality. Moreover, the use of different outcome measures in the reviewed studies hindered the comparison of findings across the studies. In addition to the survey modes, many other factors, such as study design, data collection settings, social norms, and participant characteristics, could have an impact on self-reports of sexual behaviors, and this was not reported in detail by many of the reviewed studies. Therefore, the review findings suggested that the ACASI mode might be a useful survey mode in administering sexual behavior surveys in Asia and sub-Saharan Africa; however, this requires more studies with comparable outcome measures and information about other factors to be taken into consideration in surveying sexual behaviors and to better understand the mode effect of ACASIs in sexual behavior surveys.

## Appendix

Multimedia Appendix 1 Supplementary material.

## Abbreviations

ACASI: audio computer-assisted self-interview
BBV: blood-borne virus
FTFI: face-to-face interview
JBI: Joanna Briggs Institute
OR: odds ratio
PRISMA: Preferred Reporting Items for Systematic Reviews and Meta-Analyses
PROSPERO: International Prospective Register of Systematic Reviews
RSID: rapid stain identification of human semen
STD: sexually transmitted disease
STI: sexually transmitted infection
WHO: World Health Organization
