# Supplementary material for: Comparison of the ACASI Mode to Other Survey Modes in Sexual Behavior Surveys in Asia and Sub-Saharan Africa: Systematic Literature Review
Source: J Med Internet Res. 2022 May 31;24(5):e37356. doi: 10.2196/37356 (PMC9198818; doi:10.2196/37356)
Supplement: Multimedia Appendix 1 [file jmir_v24i5e37356_app1.docx]

Multimedia Appendix 1: Supplementary Material


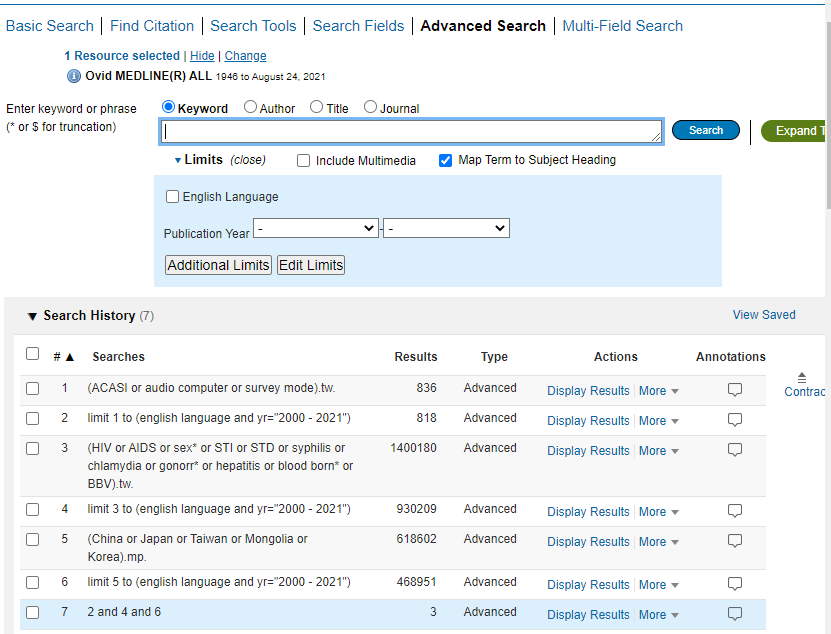


### **Figure S1. One of the search strategies used to search in MEDLINE database, Comparison of audio computer-assisted self-interview (ACASI) to other survey modes in sexual behavior surveys in Asia and sub-Saharan Africa: Systematic literature review**

### **Table S1. Search strategies used in the MEDLINE database, Comparison of audio computer-assisted self-interview (ACASI) to other survey modes in sexual behavior surveys in Asia and sub-Saharan Africa: Systematic literature review**

| **Sr. No.** | **Date of search** | **Title or Abstract** | **AND /OR** | **Title or Abstract** | **AND /OR** | **All fields** | **No. of results** |
| --- | --- | --- | --- | --- | --- | --- | --- |
| 1 | 24/08/2021 | (ACASI.tw. OR audio computer.tw. OR survey mode.tw.) | AND | (HIV.tw. OR AIDS.tw. OR sex*.tw. OR STI.tw. OR STD.tw. OR syphilis.tw. OR chlamydia.tw. OR gonorr*.tw. OR hepatitis.tw. OR blood born*.tw. OR BBV.tw.) | AND | (Brunei.mp. OR Burma.mp. OR Myanmar.mp. OR Cambodia.mp. OR Timor*.mp. OR Indonesia.mp. OR Laos.mp. OR Malaysia.mp. OR Philippines.mp. OR Singapore.mp. OR Thai*.mp. OR Vietnam.mp.) | 28 |
| 2 | 24/08/2021 | (ACASI.tw. OR audio computer.tw. OR survey mode.tw.) | AND | (HIV.tw. OR AIDS.tw. OR sex*.tw. OR STI.tw. OR STD.tw. OR syphilis.tw. OR chlamydia.tw. OR gonorr*.tw. OR hepatitis.tw. OR blood born*.tw. OR BBV.tw.) | AND | (China.mp. OR Japan.mp. OR Taiwan.mp. OR Mongolia.mp. OR Korea.mp.) | 3 |
| 3 | 24/08/2021 | (ACASI.tw. OR audio computer.tw. OR survey mode.tw.) | AND | (HIV.tw. OR AIDS.tw. OR sex*.tw. OR STI.tw. OR STD.tw. OR syphilis.tw. OR chlamydia.tw. OR gonorr*.tw. OR hepatitis.tw. OR blood born*.tw. OR BBV.tw.) | AND | (Afghanistan.mp. OR India.mp. OR Pakistan.mp. OR Bangladesh.mp. OR Sri Lanka.mp. OR Nepal.mp. OR Bhutan.mp. OR Maldives.mp.) | 6 |
| 4 | 24/08/2021 | (ACASI.tw. OR audio computer.tw. OR survey mode.tw.) | AND | (HIV.tw. OR AIDS.tw. OR sex*.tw. OR STI.tw. OR STD.tw. OR syphilis.tw. OR chlamydia.tw. OR gonorr*.tw. OR hepatitis.tw. OR blood born*.tw. OR BBV.tw.) | AND | (Armenia.mp. OR Azerbaijan.mp. OR Cyprus.mp. OR Georgia.mp. OR Kazakhstan.mp. OR Kyrgyz*.mp. OR Russia*.mp. OR Tajikistan.mp. OR Turkey.mp. OR Turkmenistan.mp. or Uzbekistan.mp.) | 31 |
| 5 | 24/08/2021 | (ACASI.tw. OR audio computer.tw. OR survey mode.tw.) | AND | (HIV.tw. OR AIDS.tw. OR sex*.tw. OR STI.tw. OR STD.tw. OR syphilis.tw. OR chlamydia.tw. OR gonorr*.tw. OR hepatitis.tw. OR blood born*.tw. OR BBV.tw.) | AND | (Angola.mp. OR Benin.mp. OR Botswana.mp. OR Burkina Faso.mp. OR Burundi.mp. OR Cabo Verde.mp. OR Cameroon.mp. OR Central African Republic.mp.) | 4 |
| 6 | 24/08/2021 | (ACASI.tw. OR audio computer.tw. OR survey mode.tw.) | AND | (HIV.tw. OR AIDS.tw. OR sex*.tw. OR STI.tw. OR STD.tw. OR syphilis.tw. OR chlamydia.tw. OR gonorr*.tw. OR hepatitis.tw. OR blood born*.tw. OR BBV.tw.) | AND | (Chad.mp. OR Comoros.mp. OR Democratic Republic of Congo.mp. OR Republic of the Congo.mp. OR Ivory Coast.mp. OR Equatorial Guinea.mp. OR Eritrea.mp.) | 2 |
| 7 | 24/08/2021 | (ACASI.tw. OR audio computer.tw. OR survey mode.tw.) | AND | (HIV.tw. OR AIDS.tw. OR sex*.tw. OR STI.tw. OR STD.tw. OR syphilis.tw. OR chlamydia.tw. OR gonorr*.tw. OR hepatitis.tw. OR blood born*.tw. OR BBV.tw.) | AND | (Eswatini.mp. OR Ethiopia.mp. OR Gabon.mp. OR The Gambia.mp. OR Ghana.mp. OR Guinea.mp. OR Guinea-Bissau.mp. OR Kenya.mp.) | 25 |
| 8 | 24/08/2021 | (ACASI.tw. OR audio computer.tw. OR survey mode.tw.) | AND | (HIV.tw. OR AIDS.tw. OR sex*.tw. OR STI.tw. OR STD.tw. OR syphilis.tw. OR chlamydia.tw. OR gonorr*.tw. OR hepatitis.tw. OR blood born*.tw. OR BBV.tw.) | AND | (Lesotho.mp. OR Liberia.mp. OR Madagascar.mp. OR Malawi.mp. OR Mali.mp. OR Mauritana.mp. OR Mauritius.mp. OR Mozambique.mp.) | 7 |
| 9 | 24/08/2021 | (ACASI.tw. OR audio computer.tw. OR survey mode.tw.) | AND | (HIV.tw. OR AIDS.tw. OR sex*.tw. OR STI.tw. OR STD.tw. OR syphilis.tw. OR chlamydia.tw. OR gonorr*.tw. OR hepatitis.tw. OR blood born*.tw. OR BBV.tw.) | AND | (Namibia.mp. OR Niger.mp. OR Nigeria.mp. OR Rwanda.mp. OR Sao Tome and Principe.mp. OR Senegal.mp. OR Seychelles.mp. OR Sierra Leone.mp. OR Somalia.mp.) | 0 |
| 10 | 24/08/2021 | (ACASI.tw. OR audio computer.tw. OR survey mode.tw.) | AND | (HIV.tw. OR AIDS.tw. OR sex*.tw. OR STI.tw. OR STD.tw. OR syphilis.tw. OR chlamydia.tw. OR gonorr*.tw. OR hepatitis.tw. OR blood born*.tw. OR BBV.tw.) | AND | (South Africa.mp. OR South Sudan.mp. OR Sudan.mp. OR Tanzania.mp. OR Togo.mp. OR Uganda.mp. OR Zambia.mp. OR Zimbabwe.mp.) | 52 |
| TOTAL | | | | | | | 158 |

### **Table S2. Comparison of audio computer-assisted self-interview (ACASI) to other survey modes in sexual behavior surveys in Asia and sub-Saharan Africa: Systematic literature review**

| **Author, Year** | | **Variables** | | **Results** | | | | **Reference mode** | **No. of comparisons between ACASI and other modes** |
| --- | --- | --- | --- | --- | --- | --- | --- | --- | --- |
|  |  |  |  | **Survey mode** | | **Test statistics** | **n/N** |  |  |
| (1) Had boyfriend/ girlfriend/ partner | | | | | | | | | |
| Mensch, 2003 | | Ever had a boyfriend or a girlfriend – boys, Nyeri | | ACASI  SAQ | OR 0.51***  OR 0.62*** | |  | FTFI | 1 |
|  | | Ever had a boyfriend or a girlfriend –girls, Nyeri | | ACASI  SAQ | OR 0.49***  OR 0.56*** | |  | FTFI | 1 |
|  | | Ever had a boyfriend or a girlfriend – boys, Kisumu | | ACASI  SAQ | OR 0.43***  OR 0.78 | |  | FTFI | 1 |
|  | | Ever had a boyfriend or a girlfriend –girls, Kisumu | | ACASI  SAQ | OR 0.69*  OR 0.82 | |  | FTFI | 1 |
| Potdar 2005 | | Relationship with a woman, college | | ACASI  SAQ | AOR 1.80*  AOR 1.13 | |  | FTFI | 1 |
|  | | Relationship with a woman, slum | | ACASI | AOR 0.84 | |  | FTFI | 1 |
|  | | Relationship with a man, college | | ACASI  SAQ | AOR 8.10**  AOR 4.22 | |  | FTFI | 1 |
|  | | Relationship with a man, slum | | ACASI | AOR 1.18 | |  | FTFI | 1 |
| Jaya 2008 | | Casual relationship with opposite sex, boys | | ACASI  FTFI | 7.6%  7.6% | | 22/290  22/290 | - | 1 |
|  | | Casual relationship with opposite sex, girls | | ACASI  FTFI | 3.9%  3.4% | | 9/233  8/233 | - | 1 |
| Kelly, 2013 | | ever had boyfriend/ girlfriend, male | | ACASI | AOR 0.40 (0.27-0.57)*** | |  | FTFI | 1 |
|  | | ever had boyfriend/ girlfriend, female | | ACASI | AOR 0.71 (0.49-1.02) | |  | FTFI | 1 |
| (2) No partner | | | | | | | | | |
| Gorbach, 2013 | | No partner past 3 months | | ACASI  FTFI | 12.3%*  4.8%* | | 72/585  28/585 | - | 1 |
| (3) Ever had sex | | | | | | | | | |
| Mensch, 2003 | | Ever had sex - boys, Nyeri | | ACASI  FTFI | OR 0.81  OR 1.60*** | |  | SAQ | 1 |
|  | | Ever had sex – girls, Nyeri | | ACASI  FTFI | OR 0.87  OR 1.79*** | |  | SAQ | 1 |
|  | | Ever had sex – boys, Kisumu | | ACASI  FTFI | OR 0.55***  OR 1.18 | |  | SAQ | 1 |
|  | | Ever had sex – girls, Kisumu | | ACASI  FTFI | OR 0.84  OR 1.22 | |  | SAQ | 1 |
| Hewett, 2004 | | Ever had sex | | ACASI | OR 1.22 | |  | FTFI | 1 |
| Potdar, 2005 | | Vaginal intercourse with a woman - college youth | | ACASI  SAQ | AOR 1.99  AOR 1.17 | |  | FTFI | 1 |
|  | | Vaginal intercourse with a woman - slum youth | | ACASI | AOR 0.23*** | |  | FTFI | 1 |
|  | | Anal intercourse with a woman – slum youth | | ACASI | AOR 3.87*** | |  | FTFI | 1 |
|  | | Anal intercourse with a man - college youth | | ACASI  SAQ | AOR 4.58  AOR 4.55 | |  | FTFI | 1 |
|  | | Anal intercourse with a man - slum youth | | ACASI | AOR 0.59 | |  | FTFI | 1 |
| Griensven, 2006 | | Had sexual contact | | ACASI  SAQ  FTFI  PASI | 53.8%  41.2%  64.7%  43.3% | | 175/325  129/313  205/317  142/328 | PASI Vs ACASI | 1 |
|  | | Had sexual intercourse | | ACASI  SAQ  FTFI  PASI | 55.8%***  50.8%  56.8%  58.5%*** | | 183/325  159/313  180/317  192/328 | PASI Vs ACASI | 1 |
| Le, 2006 | | Had sexual intercourse - male | | ACASI  SAQ | OR 1.16  OR 0.70 | |  | FTFI | 1 |
|  | | Had sexual intercourse - female | | ACASI  SAQ | OR 1.17  OR 1.55* | |  | FTFI | 1 |
|  | | Had sexual intercourse – 15 to 19 year old | | ACASI  SAQ | OR 2.79*  OR 1.36 | |  | FTFI | 1 |
|  | | Had sexual intercourse – 20 to 24 year old | | ACASI  SAQ | OR 1.09  OR 1.02 | |  | FTFI | 1 |
|  | | Had sexual intercourse – single | | ACASI  SAQ | OR 1.77*  OR 0.30* | |  | FTFI | 1 |
| Li, 2007 | | Had sexual intercourse | | ACASI  CAPI | 60.9%***  79.7%*** | | 121/199  159/199 | - | 1 |
| NIMH, 2007 | | Ever had sex - China | | ACASI  CAPI | Same on both ACASI and CAPI (ACASI first) – 78%**  Same on both ACASI and CAPI (CAPI first) – 74%  ‘yes’ for ACASI and ‘no’ for CAPI (ACASI first) – 3%  ‘yes’ for ACASI and ‘no’ for CAPI (CAPI first) – 2%  ‘yes’ for CAPI and ‘no’ for ACASI (ACASI first) – 19%  ‘yes’ for CAPI and ‘no’ for ACASI (CAPI first) – 23% | | 78/100  73/99  3/100  2/99  19/100  23/99 | - | 1 |
| Jaya, 2008 | | Ever had sex with opposite sex – boys | | ACASI  FTFI | 26.9%  21.4% | | 78/290  62/290 | - | 1 |
|  | | Ever had sex with opposite sex – girls | | ACASI  FTFI | 3.4%  1.3% | | 8/233  3/233 | - | 1 |
| Mensch, 2008 | | Had sex – Malawi | | ACASI  FTFI | 34.8%  47.9%** | | 73/211  113/236 | - | 1 |
|  | | Had sex - Kenya | | ACASI  FTFI | 42.8%  48.3% | | 129/302  168/348 | - | 1 |
| Langhaug, 2011 | | Had sex | | ACASI  SAQ  Audio-SAQ  ICVI | 10.9%  10.4%  14.5%  11.9% | | 41/381  39/373  54/376  43/365 | - | 1 |
| Kelly, 2013 | | Had sex - male | | ACASI | AOR 0.66 (0.42-1.04) | |  | FTFI | 1 |
|  | | Had sex – female | | ACASI | AOR 1.13 (0.79-1.61) | |  | FTFI | 1 |
| Kelly, 2014 | | Had sex | | ACASI  FTFI  CAPI | 92.0% (FTF Vs ACASI)  87.9%  86.0%* (CAPI Vs ACASI) | | 310/338  320/365  273/317 | - | 2 |
|  | | Sex in past 2 days | | ACASI  FTFI  CAPI | 29.7%  25.8%  26.7% | | 100/338  94/365  85/317 | - | 1 |
| (4) Age at first intercourse | | | | | | | | | |
| Langhaug, 2011 | | Age at first intercourse | | ACASI  SAQ  Audio-SAQ  ICVI | Mean 15.2 (14.1 – 16.2)*  Mean 15.9 (14.3 – 17.5)*  Mean 16.7 (16.0 – 17.4)*  Mean 16.9 (16.1 – 17.8)* | |  | - | 3 |
| Kelly, 2013 | | Age at first sex, male | | ACASI  FTFI | Mean 13.7  Mean 13.7 | |  | - | 1 |
|  | | Age at first sex, female | | ACASI  FTFI | Mean 14.8  Mean 15.1 | |  |  | 1 |
| (5) Multiple sexual partner | | | | | | | | | |
| Mensch, 2003 | | More than one sexual partner - boys | | ACASI  SAQ | OR 1.28  OR 1.02 | |  | FTFI | 1 |
|  | | More than one sexual partner - girls | | ACASI  SAQ | OR 2.35***  OR 0.72 | |  | FTFI | 1 |
| Potdar, 2005 | | Two and more sexual partners – college youth | | ACASI  SAQ | AOR 1.55  AOR 0.81 | |  | FTFI | 1 |
|  | | Two and more sexual partners – slum youth | | ACASI | AOR 1.02 | |  | FTFI | 1 |
| Le, 2006 | | No. of sexual partners - male | | ACASI  SAQ  FTFI | Mean 3.46 (2.60-4.32)*  Mean 2.26 (0.93-3.59)  Mean 1.46 (1.20-1.72) | |  | ACASI | 2 |
|  | | No. of sexual partners - female | | ACASI  SAQ | Mean 1.92 (1.45-2.39)*  Mean 1.02 (0.98-1.05)  Mean 1.06 (0.94-1.18) | |  | ACASI | 2 |
|  | | No. of sexual partners - married | | ACASI  SAQ  FTFI | Mean 1.69 (1.11-2.26)*  Mean 1.02 (0.98-1.05)  Mean 1.03 (0.97-1.09) | |  | ACASI | 2 |
|  | | No. of sexual partners - single | | ACASI  SAQ  FTFI | Mean 3.46 (2.76-4.15)*  Mean 2.30 (1.04-3.56)  Mean 1.63 (1.34-1.92) | |  | ACASI | 2 |
|  | | No. of sexual partners – 15-19 year old | | ACASI  SAQ  FTFI | Mean 2.43 (1.23-3.64)*  Mean 1.00 (1.00-1.00)  Mean 1.00 (1.00-1.00) | |  | ACASI | 2 |
|  | | No. of sexual partners – 20-24 year old | | ACASI  SAQ  FTFI | Mean 2.90 (2.25-3.55)*  Mean 1.48 (0.91-2.04)  Mean 1.28 (1.11-1.44) | |  | ACASI | 2 |
| Minnis, 2007 | | Multiple partners | | ACASI | OR 5.7 (2.1-15.2)*** | |  | FTFI | 1 |
| Li, 2007 | | More than one sexual partner - lifetime | | ACASI  CAPI | 12.2%  10.7% | | 23/199  20/199 | - | 1 |
| Edwards, 2008 | | Multiple sex partner, past 6 months | | ACASI  FTFI | 69%  69% | | 124/180  124/180 | - | 1 |
| Mensch, 2008 | | More than one sex partner, lifetime – Malawi | | ACASI  FTFI | 27.2%**  16.5% | | 57/211  39/236 | - | 1 |
|  | | More than one sex partner, lifetime – Kenya | | ACASI  FTFI | 34.9%**  20.7% | | 105/302  72/348 | - | 1 |
| Van der Elst, 2009 | | Women, casual partner, past week | | ACASI  FTFI | Median 3 (1-5)*  Median 2 (1-4)* | |  | - | 1 |
|  | | Women, new partner, past week | | ACASI  FTFI | Median 2 (1-4)***  Median 1 (0-3)*** | |  | - | 1 |
|  | | Women, casual partner, past month | | ACASI  FTFI | Median 2 (1-7)  Median 1 (0-4) | |  | - | 1 |
|  | | Women, new partner, past month | | ACASI  FTFI | Median 2 (0-4)  Median 1 (0-2) | |  | - | 1 |
|  | | Women, group sex, past 3 months | | ACASI  FTFI | 23.0%  18.7% | | 32/139  26/139 | - | 1 |
|  | | Men, casual partner, past week | | ACASI  FTFI | Median 2 (1-3)***  Median 1 (0-3)*** | |  | - | 1 |
|  | | Men, new partner, past week | | ACASI  FTFI | Median 1 (1-3)***  Median 1 (0-2)*** | |  | - | 1 |
|  | | Men, casual partner, past month | | ACASI  FTFI | Median 3 (1-5)  Median 1 (0-3) | |  | - | 1 |
|  | | Men, new partner, past month | | ACASI  FTFI | Median 1 (0-3)  Median 0 (0-2) | |  | - | 1 |
|  | | Men, group sex, past 3 months | | ACASI  FTFI | 21.6%***  13.5%*** | | 56/259  35/259 | - | 1 |
| Mensch, 2011 | | More than 1 sex partner, last month (enrolment) | | ACASI  FTFI | 13.8%**  1.7%** | | (N=414-424)  (N=418-425) | - | 1 |
| Langhaug, 2011 | | Mean number of lifetime sex partners | | ACASI  SAQ  Audio-SAQ  ICVI | Mean 2.4 (1.8 – 2.9)  Mean 1.7 (1.3 – 2.2)  Mean 1.8 (1.3 – 2.4)  Mean 1.5 (1.2 – 1.9) | |  | - | 3 |
| Le, 2012 | | Mean number of sex partners, last 12 months, married | | ACASI  SAQ  FTFI | 1.3*  1.0*  1.2* | |  | ACASI | 2 |
|  | | Mean number of sex partners, last 12 months, unmarried | | ACASI  SAQ  FTFI | 2.3  2.1  1.6 | |  | ACASI | 2 |
|  | | Mean number of sex partners, last 12 months, male | | ACASI  SAQ  FTFI | 1.9*  1.2*  1.4* | |  | ACASI | 2 |
|  | | Mean number of sex partners, last 12 months, female | | ACASI  SAQ  FTFI | 1.1  1.0  1.1 | |  | ACASI | 2 |
|  | | Mean number of sex partners, last 12 months, urban | | ACASI  SAQ  FTFI | 1.5*  1.2*  1.2* | |  | ACASI | 2 |
|  | | Mean number of sex partners, last 12 months, rural | | ACASI  SAQ  FTFI | 1.4*  1.0*  1.1* | |  | ACASI | 2 |
| Gorbach, 2013 | | More than 1 partner, past 3 months | | ACASI  FTFI | 1.0%*  0%* | | 6/585  0/585 | - | 1 |
| Adebajo, 2014 | | Multiple female sex partners, past 2 months | | ACASI | AOR 1.4 (1.1 – 1.9)* | |  | FTFI | 1 |
|  | | Multiple male sex partners, past 2 months | | ACASI | AOR 2.1 (1.5 – 2.8)*** | |  | FTFI | 1 |
|  | | Had casual sex partners, past 2 months | | ACASI | OR 3.7 (1.2-11.4)* | |  | FTFI | 1 |
| Kelly, 2014 | | Multiple lifetime sex partners | | ACASI  FTFI  CAPI | 71.5% (FTFI Vs ACASI)  66.9%  67.8% (CAPI Vs ACASI) | | 242/338  244/365  215/317 | - | 2 |
| Desmond, 2018 | | More than one sex partner, last 3 months – women (baseline) | | ACASI  FTFI | 3.4%  6.8% | | 5/147  10/147 | - | 1 |
|  | | More than one sex partner, last 3 months – men (baseline) | | ACASI  FTFI | 12.9%  14.4% | | 18/139  20/139 | - | 1 |
|  | | More than one sex partner, lifetime – women (baseline) | | ACASI  FTFI | 68.7%  67.3% | | 101/147  99/147 | - | 1 |
|  | | More than one sex partner, lifetime – men (baseline) | | ACASI  FTFI | 81.3%  81.3% | | 113/139  113/139 | - | 1 |
| (6) Types of sexual partner | | | | | | | | | |
| Hewett, 2004 | | Boyfriend | | ACASI | OR 1.16 | |  | FTFI | 1 |
|  | | Friend, schoolmate, acquaintance | | ACASI | OR 6.26*** | |  | FTFI | 1 |
|  | | Relative | | ACASI | OR 42.60*** | |  | FTFI | 1 |
|  | | 10 or more years older | | ACASI | OR 3.55*** | |  | FTFI | 1 |
| Mensch, 2008 | | Boyfriend - Malawi | | ACASI  FTFI | 21.0%  30.9%** | | 44/211  73/236 | - | 1 |
|  | | Boyfriend - Kenya | | ACASI  FTFI | 40.1%  45.7% | | 121/302  159/348 | - | 1 |
|  | | Expected spouse - Malawi | | ACASI  FTFI | 29.4%  27.5% | | 96/211  65/236 | - | 1 |
|  | | Acquaintance – Malawi | | ACASI  FTFI | 17.1%**  6.8% | | 36/211  16/236 | - | 1 |
|  | | Acquaintance – Kenya | | ACASI  FTFI | 31.9%**  9.8% | | 96/302  34/348 | - | 1 |
|  | | Family member – Malawi | | ACASI  FTFI | 7.1%  1.3% | | 15/211  3/236 | - | 1 |
|  | | Family member – Kenya | | ACASI  FTFI | 20.9%**  1.0% | | 63/302  3/348 | - | 1 |
|  | | Teacher – Malawi | | ACASI  FTFI | 1.4%  1.0% | | 3/211  3/236 | - | 1 |
|  | | Employer – Malawi | | ACASI  FTFI | 1.9%  1.0% | | 4/211  2/236 | - | 1 |
|  | | Composite: Any partner – Malawi | | ACASI  FTFI | 50.7%  47.4% | | 107/211  112/236 | - | 1 |
|  | | Composite: Any partner – Kenya | | ACASI  FTFI | 61.0%*  48.0% | | 184/302  167/348 | - | 1 |
|  | | Composite: sex or sex with any partner - Malawi | | ACASI  FTFI | 57.8%*  48.3% | | 122/211  114/236 | - | 1 |
|  | | Composite: sex or sex with any partner - Kenya | | ACASI  FTFI | 67.5%**  48.9% | | 204/302  170/348 | - | 1 |
| Kelly, 2013 | | Boyfriend/ girlfriend, male | | ACASI  FTFI | 40.2%***  62.6% | | 444/1104  122/195 | - | 1 |
|  | | Hit and run, male | | ACASI  FTFI | 12.8%**  20.5% | | 141/1104  40/195 | - | 1 |
|  | | Relative, male | | ACASI  FTFI | 8.2%*  3.1% | | 91/1104  6/195 | - | 1 |
|  | | Teacher, male | | ACASI  FTFI | 2.6%*  0% | | 29/1104  0/195 | - | 1 |
|  | | Other, male | | ACASI  FTFI | 12.3%  7.7% | | 136/1104  15/195 | - | 1 |
|  | | Any partner, male | | ACASI  FTFI | 51.6%***  70.3% | | 570/1104  137/195 | - | 1 |
|  | | Stigmatised partner, male | | ACASI  FTFI | 10.0%**  3.1% | | 110/1104  6/195 | - | 1 |
|  | | Boyfriend/ girlfriend, female | | ACASI  FTFI | 31.3%  35.3% | | 202/646  41/116 | - | 1 |
|  | | Hit and run, female | | ACASI  FTFI | 4.2%  2.6% | | 27/646  3/116 | - | 1 |
|  | | Relative, female | | ACASI  FTFI | 3.7%*  0% | | 24/646  0/116 | - | 1 |
|  | | Teacher, female | | ACASI  FTFI | 1.7%  0% | | 11/646  0/116 | - | 1 |
|  | | Other, female | | ACASI  FTFI | 4.5%  1.7% | | 29/646  2/116 | - | 1 |
|  | | Any partner, female | | ACASI  FTFI | 35.6%  39.7% | | 230/646  46/116 | - | 1 |
|  | | Stigmatised partner, female | | ACASI  FTFI | 4.8%*  0% | | 31/646  0/116 | - | 1 |
| (7) Pre-marital sex | | | | | | | | | |
| Le, 2006 | | Rate of pre-marital sex – all males | | ACASI  SAQ  FTFI | 18.3%  10.0%  15.2% | | (N-not reported) | ACASI | 2 |
|  | | Rate of pre-marital sex – all females | | ACASI  SAQ  FTFI | 7.4%  6.3%  5.2% | |  | ACASI | 2 |
|  | | Rate of pre-marital sex – unmarried males | | ACASI  SAQ  FTFI | 17.1%  8.6%  12.7% | |  | ACASI | 2 |
|  | | Rate of pre-marital sex – unmarried females | | ACASI  SAQ  FTFI | 4.5%  4.2%  1.7% | |  | ACASI | 2 |
| Le, 2012 | | Had premarital sex | | ACASI  SAQ  FTFI | 20.4%*  11.1%  11.4% | | 314/1540  171/1540  176/1540 | ACASI | 2 |
| (8) Transactional sex | | | | | | | | | |
| Griensven, 2006 | | Sold sex | | ACASI  SAQ  FTFI  PASI | 5.5%***  7.7%  0.9%  6.7%*** | | 18/325  24/313  3/317  22/328 | PASI Vs ACASI | 1 |
|  | | Bought sex | | ACASI  SAQ  FTFI  PASI | 7.1%***  3.5%  2.5%  8.2%*** | | 23/325  11/313  8/317  27/328 | PASI Vs ACASI | 1 |
| Le, 2006 | | Sex with a sex worker – 15 to 19 year old | | ACASI  SAQ | OR 4.88  OR 1.05 | |  | FTFI | 1 |
|  | | Sex with a sex worker – 20 to 24 year old | | ACASI  SAQ | OR 2.05*  OR 0.23* | |  | FTFI | 1 |
| Edwards, 2008 | | Buy sex | | ACASI  FTFI | 19%  19% | | 34/180  34/180 | - | 1 |
|  | | Sell sex | | ACASI  FTFI | 12%*  9%* | | 22/180  16/180 | - | 1 |
| Van der Elst, 2009 | | Received cash for sex in past 3 months – women | | ACASI  FTFI | 87.1%*  95.7%* | | 121/139  133/139 | - | 1 |
|  | | Paid for sex in past 3 months - women | | ACASI  FTFI | 49.3%**  5.8%** | | 68/139  8/139 | - | 1 |
|  | | Received cash for sex in past 3 months – men | | ACASI  FTFI | 64.5%**  71.4%** | | 167/259  185/259 | - | 1 |
|  | | Paid for sex in past 3 months - men | | ACASI  FTFI | 45.2%  39.4% | | 117/259  102/259 | - | 1 |
| Le, 2012 | | Had sex with a sex worker | | ACASI  SAQ  FTFI | 4.3%  5.5%  2.3% | | 66/1540  85/1540  35/1540 | ACASI | 2 |
| Kelly, 2014 | | Transactional sex in past 12 months | | ACASI  FTFI  CAPI | 19.4%* (FTFI Vs ACASI)  12.0%  16.4% (CAPI Vs ACASI) | | 66/338  44/365  52/317 | - | 2 |
| (9) Sex with a stranger | | | | | | | | | |
| Mensch, 2003 | | Sex with a stranger – boys, Kisumu | | ACASI  SAQ | OR 2.42**  OR 1.43 | |  | FTFI | 1 |
|  | | Sex with a stranger – girls, Kisumu | | ACASI  SAQ | OR 4.25***  OR 1.24 | |  | FTFI | 1 |
| Hewett, 2004 | | Sexual intercourse with a stranger | | ACASI | OR 3.45*** | |  | FTFI | 1 |
| Potdar, 2005 | | Non-regular partner, college youth | | ACASI  SAQ | AOR 1.52  AOR 1.12 | |  | FTFI | 1 |
|  | | Non-regular partner, slum youth | | ACASI | AOR 0.77 | |  | FTFI | 1 |
| Mensch, 2008 | | Sex with stranger, Malawi | | ACASI  FTFI | 3.3%  2.5% | | 7/211  6/236 | - | 1 |
|  | | Sex with stranger, Kenya | | ACASI  FTFI | 13.9%**  3.7% | | 42/302  13/348 | - | 1 |
| (10) Forced sex | | | | | | | | | |
| Mensch, 2003 | | Tricked/ coerced/ forced into sex – boys, Kisumu | | ACASI  SAQ | OR 2.40***  OR 2.33*** | |  | FTFI | 1 |
|  | | Tricked/ coerced/ forced into sex – girls, Kisumu | | ACASI  SAQ | OR 3.35***  OR 1.89** | |  | FTFI | 1 |
| Hewett, 2004 | | Coerced sex - tricked | | ACASI | OR 3.62*** | |  | FTFI | 1 |
|  | | Coerced sex – locked in a room | | ACASI | OR 2.74*** | |  | FTFI | 1 |
|  | | Coerced sex – physically forced | | ACASI | OR 2.73*** | |  | FTFI | 1 |
| Potdar, 2005 | | Coercive sex – college youth | | ACASI  SAQ | AOR 11.35**  AOR 2.13 | |  | FTFI | 1 |
|  | | Coercive sex – slum youth | | ACASI | AOR 1.99 | |  | FTFI | 1 |
| Jaya, 2008 | | Forced sexual intercourse – boys | | ACASI  FTFI | 11.4%  7.9% | | 33/290  23/290 | - | 1 |
|  | | Forced sexual intercourse – girls | | ACASI  FTFI | 2.6%  1.3% | | 6/233  3/233 | - | 1 |
| Van der Elst, 2009 | | Violence, been raped in past 3 months – women | | ACASI  FTFI | 6.9%  4.4% | | 9/139  6/139 | - | 1 |
|  | | Violence, been raped in past 3 months – men | | ACASI  FTFI | 8.9%**  3.9%** | | 23/259  10/259 | - | 1 |
| Mensch, 2011 | | Forced to have sex, last month (enrolment) | | ACASI  FTFI | 7.1%**  2.8%** | | (N=414-424)  (N=418-425) | - | 1 |
| Kelly, 2014 | | Forced sex | | ACASI  FTFI  CAPI | 39.2%* (FTFI Vs ACASI)  30.6%  34.3% (CAPI Vs ACASI) | | 132/338  111/365  109/317 | - | 2 |
| (11) Oral sex | | | | | | | | | |
| Potdar, 2005 | | Oral sex, with a man – college youth | | ACASI  SAQ | AOR 7.75**  AOR 3.16 | |  | FTFI | 1 |
|  | | Oral sex, with a man – slum youth | | ACASI | AOR 3.20* | |  | FTFI | 1 |
|  | | Oral sex, with a woman – college youth | | ACASI  SAQ | AOR 2.08*  AOR 0.95 | |  | FTFI | 1 |
|  | | Oral sex, with a woman – slum youth | | ACASI | AOR 2.40** | |  | FTFI | 1 |
| Griensven, 2006 | | Sexual contact involved oral sex | | ACASI  SAQ  FTFI  PASI | 42.9%  42.6%  13.2%  37.3% | | 75/175  55/129  27/205  53/142 | PASI Vs ACASI | 1 |
| (12) Anal sex | | | | | | | | | |
| Potdar, 2005 | | Anal intercourse with a woman - college | | ACASI  SAQ | 1.3%  0.3% | |  | FTFI | 1 |
|  | | Anal intercourse with a woman - slum | | ACASI | AOR 3.87*** | |  | FTFI | 1 |
| Van der Elst 2009 | | Women, anal sex, past 3 months | | ACASI  FTFI | 39.6%  43.9% | | 55/139  61/139 | - | 1 |
|  | | Men, anal sex, past 3 months | | ACASI  FTFI | 78.0%***  92.7%*** | | 202/259  240/259 | - | 1 |
|  | | Men, anal sex, insertive | | ACASI  FTFI | 63.3%***  72.6%*** | | 164/259  188/259 | - | 1 |
|  | | Men, anal sex, receptive | | ACASI  FTFI | 44.8%***  57.5%*** | | 116/259  149/259 | - | 1 |
|  | | Men, anal sex, both insertive and receptive | | ACASI  FTFI | 29.7%**  37.5%** | | 77/259  97/259 | - | 1 |
| Mensch, 2011 | | Anal sex, last month (enrolment) | | ACASI  FTFI | 7.8%**  1.9%** | | (N=414-424)  (N=418-425) | - | 1 |
| Gorbach, 2013 | | Anal intercourse, past 3 months | | ACASI  FTFI | 5.0%*  0.2%* | | 29/585  1/585 | - | 1 |
| Adebajo, 2014 | | Anal sex, with women, last 2 months | | ACASI | AOR 13.1 (7.9 – 21.7)*** | |  | FTFI | 1 |
| (13) Masturbation | | | | | | | | | |
| Potdar, 2005 | | Masturbation – college youth | | ACASI  SAQ | AOR 1.28  AOR 1.61 | |  | FTFI | 1 |
|  | | Masturbation – slum youth | | ACASI | AOR 22.53*** | |  | FTFI | 1 |
| (14) Douching | | | | | | | | | |
| Mensch, 2011 | | Washed vagina after sex – last sex (enrolment) | | ACASI  FTFI | 61.1%**  25.8%** | | (N=414-424)  (N=418-425) | - | 1 |
|  | | Washed vagina before sex – last sex (Month 1) | | ACASI  FTFI | 31.4%**  3.0%** | | (N=414-424)  (N=418-425) | - | 1 |
|  | | Inserted something in vagina (to clean, dry, tighten) after inserted gel but before sex, last sex (Month 1) | | ACASI  FTFI | 9.5%**  2.4%** | | (N=414-424)  (N=418-425) | - | 1 |
| (15) Homosexuality | | | | | | | | | |
| Edwards, 2008 | | Same sex partner | | ACASI  FTFI | 9%  7% | | 16/180  13/180 | - | 1 |
| Jaya, 2008 | | Ever had sex with same sex - boys | | ACASI  FTFI | 6.2%  6.6% | | 18/290  19/290 | - | 1 |
|  | | Ever had sex with same sex - girls | | ACASI  FTFI | 1.4%  0.4% | | 3/233  1/233 | - | 1 |
| Van der Elst, 2009 | | Women with woman partner only | | ACASI  FTFI | 0.9%  0% | | 1/139  0/139 | - | 1 |
|  | | Men with man partner only | | ACASI  FTFI | 30.1%  32.2% | | 71/259  76/259 | - | 1 |
| Adebajo, 2014 | | Self-reported homosexuality | | ACASI | AOR 3.3 (2.4 – 4.6)*** | |  | FTFI | 1 |
| (16) Condom use/ Gel use | | | | | | | | | |
| Potdar, 2005 | | Condom use - college | | ACASI  SAQ | AOR 2.09  AOR 2.07 | |  | FTFI | 1 |
|  | | Condom use - slum | | ACASI | AOR 1.04 | |  | FTFI | 1 |
| Le, 2006 | | Rate of feeling confident to get condom - confident, male | | ACASI  SAQ  FTFI | 36.2%  44.3%  54.5% | | (N-not reported) | ACASI | 2 |
|  | | Rate of feeling confident to get condom - confident, female | | ACASI  SAQ  FTFI | 28.8%  37.9%  43.0% | |  | ACASI | 2 |
| Minnis, 2007 | | 100% male condom use | | ACASI | OR 1.1 (0.9-1.4) | |  | FTFI | 1 |
| Edwards, 2008 | | Condom use, past 6 months | | ACASI  FTFI | 56%  60% | | 101/180  108/180 | - | 1 |
|  | | Number of unprotected vaginal sex, primary partner, past 3 months | | ACASI  FTFI | Mean (SD) 26.5 (23.6)  Mean (SD) 26.2 (23.7) | |  | - | 1 |
|  | | Number of unprotected vaginal sex, secondary partner, past 3 months | | ACASI  FTFI | Mean (SD) 10.0 (14.7)  Mean (SD) 9.1 (12.2) | |  | - | 1 |
| Van der Elst, 2009 | | Condom use, always, regular partner, past week, women | | ACASI  FTFI | 46.0%  48.2% | | 64/139  67/139 | - | 1 |
|  | | Condom use, always, casual partner, past week, women | | ACASI  FTFI | 55.6%  57.6% | | 55/139  57/139 | - | 1 |
|  | | Condom use, always, anal sex, past 3 months, women | | ACASI  FTFI | 18.5%  24.6% | | 10/139  15/139 | - | 1 |
|  | | Condom use, always, regular partner, past week, men | | ACASI  FTFI | 37.7%**  23.8%** | | 46/259  29/259 | - | 1 |
|  | | Condom use, always, casual partner, past week, men | | ACASI  FTFI | 41.3%  35.5% | | 57/259  49/259 | - | 1 |
|  | | Condom use, always, anal sex, past 3 months, men | | ACASI  FTFI | 15.4%  19.3% | | 28/259  43/259 | - | 1 |
| Mensch, 2011 | | Always use condoms (Enrolment) | | ACASI  FTFI | 25.2%  24.5% | | (N=414-424)  (N=418-425) | - | 1 |
|  | | Used condom, last sex (Enrolment) | | ACASI  FTFI | 38.5%  42.2% | |  | - | 1 |
|  | | Used gel, last sex (Month 1) | | ACASI  FTFI | 79.3%  81.7% | |  | - | 1 |
|  | | Used condom and gel, last sex (Month 1) | | ACASI  FTFI | 63.7%*  55.1%* | |  | - | 1 |
|  | | Sex without gel, last month (Month 1) | | ACASI  FTFI | 59.7%**  40.8%** | |  | - | 1 |
|  | | Squeezed gel into vagina without having sex, last month (Month 1) | | ACASI  FTFI | 20.6%**  12.8%** | |  | - | 1 |
|  | | Told partners about gel (Month 1) | | ACASI  FTFI | 75.7%**  86.7%** | |  | - | 1 |
| Langhaug, 2011 | | Condom, last intercourse | | ACASI  SAQ  Audio-SAQ  ICVI | 39.5%  45.7%  59.5%  46.2% | | 150/381  170/373  224/376  169/365 | ACASI | 3 |
| Gorbach, 2013 | | Condom use, last sex | | ACASI  FTFI | 60.9%*  65.5%* | | 339/557  365/557 | - | 1 |
|  | | Gel use, last sex | | ACASI  FTFI | 73.5%*  77.2%* | | 303/412  318/412 | - | 1 |
| Adebajo, 2014 | | Unprotected anal sex, with men, last sex | | ACASI | AOR 0.9 (0.7 – 1.2) | |  | FTFI | 1 |
|  | | Unprotected anal sex, with women, last sex | | ACASI | AOR 2.1 (1.1 - 4.1)* | |  | FTFI | 1 |
| Kelly, 2014 | | Condom use, past 2 days | | ACASI  FTFI  CAPI | 30.4%*** (FTFI Vs ACASI)  4.9%  8.7%*** (CAPI Vs ACASI) | | 103/338  18/365  28/317 | - | 2 |
|  | | Unprotected sex, past 2 days | | ACASI  FTFI  CAPI | 20.7% (FTFI Vs ACASI)  24.5%  24.4% (CAPI Vs ACASI) | | 70/338  89/365  77/317 | - | 2 |
| Desmond 2018 | | Condom use, last sex - woman | | ACASI  FTFI | 19.7%  20.4% | | 29/147  30/147 | - | 1 |
|  | | Condom use, last sex - man | | ACASI  FTFI | 30.9%  30.2% | | 42/136  42/139 | - | 1 |
| (17) Contraception42/139 | | | | | | | | | |
| Griensven, 2006 | | Used contraception | | ACASI  SAQ  FTFI  PASI | 36.1%***  45.3%  73.9%  46.9%*** | | 66/183  72/159  133/180  90/192 | PASI Vs ACASI | 1 |
| (18) STI symptoms | | | | | | | | | |
| Mensch, 2003 | | Ever had an STI, boys, Nyeri | | ACASI  SAQ | OR 0.87  OR 1.54 | |  | FTFI | 1 |
|  | | Ever had an STI, girls, Nyeri | | ACASI  SAQ | OR 1.67  OR 2.81** | |  | FTFI | 1 |
|  | | Ever had an STI, boys, Kisumu | | ACASI  SAQ | OR 2.30**  OR 2.49** | |  | FTFI | 1 |
|  | | Ever had an STI, girls, Kisumu | | ACASI  SAQ | OR 2.52**  OR 1.44 | |  | FTFI | 1 |
| Griensven, 2006 | | Ever had genital sore or ulcer | | ACASI  SAQ  FTFI  PASI | 8.0%***  5.8%  2.5%  6.7%*** | | 26/325  18/313  8/317  22/328 | PASI Vs ACASI | 1 |
| Li, 2007 | | Ever had skin problems | | ACASI  CAPI | 18.6%  22.6% | | 37/199  45/199 | - | 1 |
|  | | Ever had abnormal discharge | | ACASI  CAPI | 8.0%  7.0% | | 16/199  14/199 | - | 1 |
| Minnis, 2007 | | STI diagnosed | | ACASI | OR 0.8 (0.5-1.4) | |  | FTFI | 1 |
| Edwards, 2008 | | Ever had an STI | | ACASI  FTFI | 43%  43% | | 77/180  77/180 | - | 1 |
| Adebajo, 2014 | | Had STI symptoms in the past 12 months | | ACASI | AOR 2.9 (2.1 – 4.1)*** | |  | FTFI | 1 |
| (19) HIV testing | | | | | | | | | |
| Potdar, 2005 | | Ever tested/ treated for HIV/AIDS – college | | ACASI  SAQ | AOR 1.53  AOR 1.99 | |  | FTFI | 1 |
|  | | Ever tested/ treated for HIV/AIDS – slum | | ACASI | AOR 3.07** | |  | FTFI | 1 |
| Edwards, 2008 | | prior to hospitalization, ever tested for HIV | | ACASI  FTFI | 84%*  89%* | | 151/180  160/180 | - | 1 |
| Adebajo, 2014 | | Ever tested for HIV | | ACASI | AOR 1.2 (0.8 – 1.6) | |  | FTFI | 1 |
| (20) HIV result disclosure | | | | | | | | | |
| Edwards, 2008 | | told anyone you are HIV infected | | ACASI  FTFI | 95.2%  95.2% | | 20/21  20/21 | - | 1 |
|  | | disclosed HIV status to family | | ACASI  FTFI | 95.2%  95.2% | | 20/21  20/21 | - | 1 |
|  | | disclosed HIV status to friends | | ACASI  FTFI | 57.1%  61.9% | | 12/21  13/21 | - | 1 |
|  | | disclosed HIV status to any sex partners in past 6 months | | ACASI  FTFI | 52.3%  66.6% | | 11/21  14/21 | - | 1 |
|  | | disclosed HIV status to all sex partners in past 6 months | | ACASI  FTFI | 33.3%**  42.8%** | | 7/21  9/21 | - | 1 |
| (21) Concerns of getting or having HIV | | | | | | | | | |
| Mensch, 2003 | | Suspect/ worried of having HIV/ AIDS – boys, Kisumu | | ACASI  SAQ | OR 1.28  OR 0.74 | |  | FTFI | 1 |
|  | | Suspect/ worried of having HIV/ AIDS – girls, Kisumu | | ACASI  SAQ | OR 1.28  OR 0.99 | |  | FTFI | 1 |
| (22) Family history of HIV/AIDS | | | | | | | | | |
| Mensch, 2003 | | Family member died of AIDS – boys, Kisumu | | ACASI  SAQ | OR 2.55**  OR 1.96 | |  | FTFI | 1 |
|  | | Family member died of AIDS – girls, Kisumu | | ACASI  SAQ | OR 2.88***  OR 1.94* | |  | FTFI | 1 |
| (23) Comparison with biomarkers | | | | | | | | | |
| Minnis, 2009 | | No vaginal sex within the past 2 days, PSA positive | | ACASI  FTFI | 12.5%  10.9% | | 13/104  10/92 | - | 1 |
|  | | Sex protected by a male or female condom only within the past 2 days, PSA positive | | ACASI  FTFI | 33.7%  39.1% | | 35/104  36/92 | - | 1 |
| Mensch, 2011 | | Reported no sex in past 2 days, RSID-semen positive (Enrolment) | | ACASI  FTFI | 11.5%*  17.1%* | | 43/373  66/387 | - | 1 |
|  | | Reported unprotected sex in past 2 days, RSID-semen positive (Enrolment) | | ACASI  FTFI | 12.9%**  6.7%** | | 48/373  26/387 | - | 1 |
| Kelly, 2014 | | Percent who report never having had sex among those who HSV2 test positive | | ACASI  FTFI  CAPI | 4.9% (ACASI Vs FTFI)  5.6%  4.5% | | 7/142  8/144  7/156 | - | 1 |
|  | | Percent reporting no unprotected sex in past 2 days among those who RSID-semen test positive | | ACASI  FTFI  CAPI | 67.1%* (ACASI Vs FTFI)  48.7%  55.1% | | 51/76  38/78  49/89 | - | 1 |
| *P* value <.05*, <.01**, <.001*** | | | | | | | | | |
| ACASI  AIDS  AOR  CAPI  FTFI  HIV  HSV2  ICVI  NR  OR  PASI  PSA  RSID - semen  SAQ  STI |  | | audio computer-assisted self-interview  Acquired Immunodeficiency Syndrome  adjusted odds ratio  computer-assisted personal interview  face-to-face interview  Human Immunodeficiency virus  Herpes simplex virus type 2  informal confidential voting interview  non-relevant  odds ratio  palmtop-assisted self-interview  prostatic specific antigen  rapid stain identification of human semen test  self-administered questionnaire  sexually transmissible infections | | | | | | |


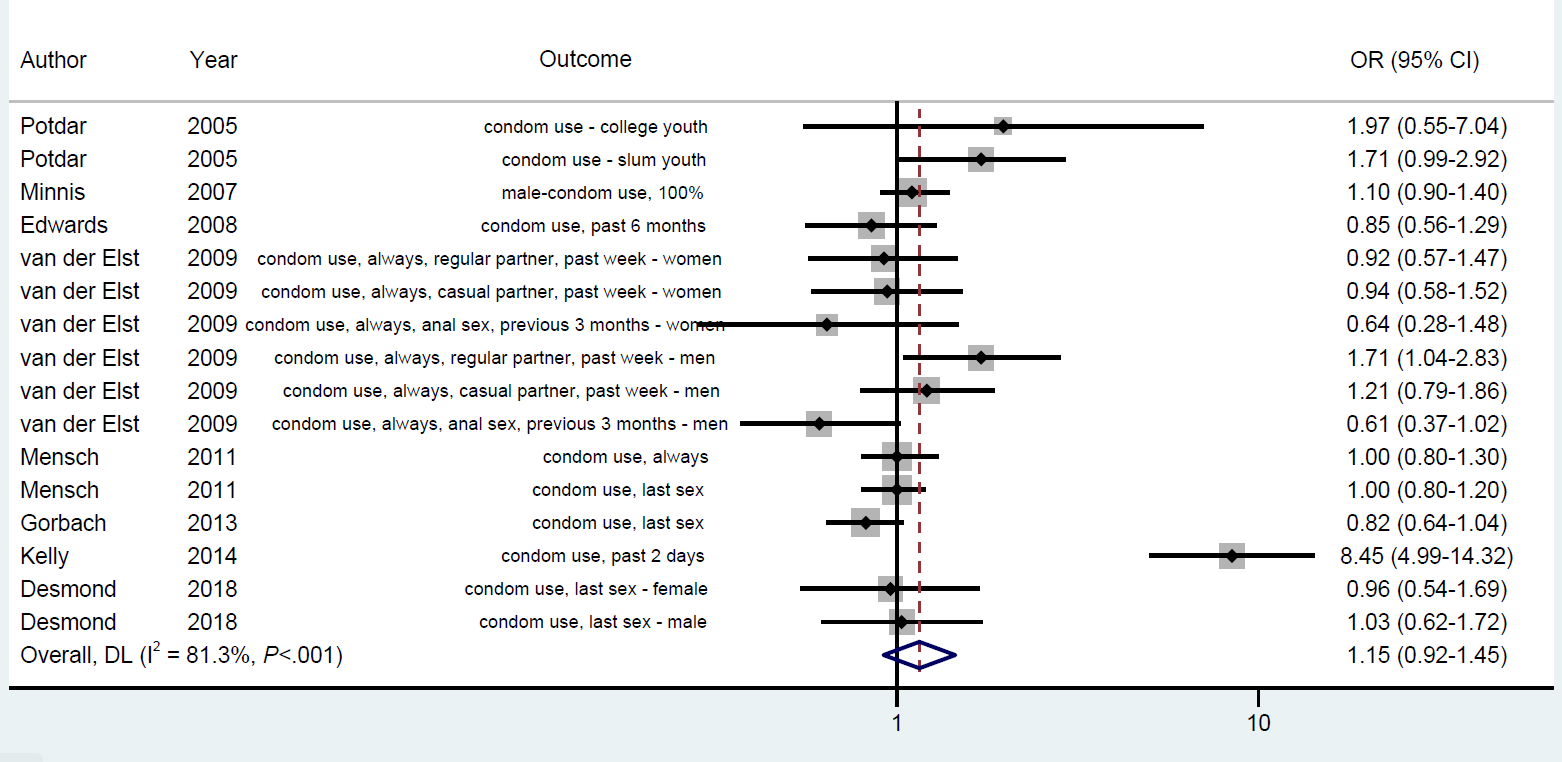


### **Figure S2. Odds ratios (ORs) of reporting “condom use” in the ACASI mode as compared to the FTFI mode. Random-effects model. ACASI: audio computer-assisted self-interview; DL: DerSimonian-Laird effect size variance estimates; FTFI: face-to-face interview.**


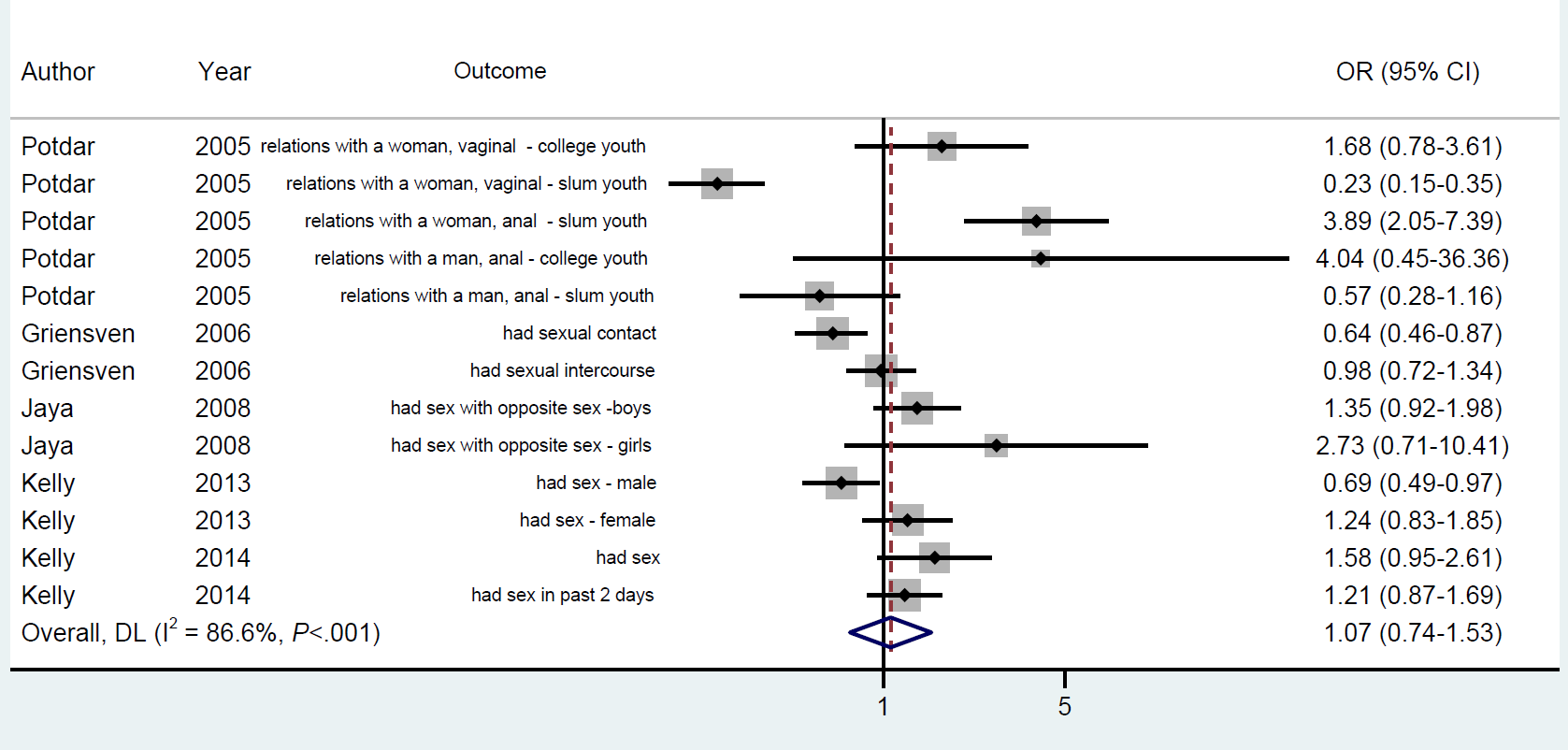


### **Figure S3. Odds ratios (ORs) of reporting “ever had sex” in the ACASI mode as compared to the FTFI mode. Random-effects model. ACASI: audio computer-assisted self-interview; DL: DerSimonian-Laird effect size variance estimates; FTFI: face-to-face interview.**


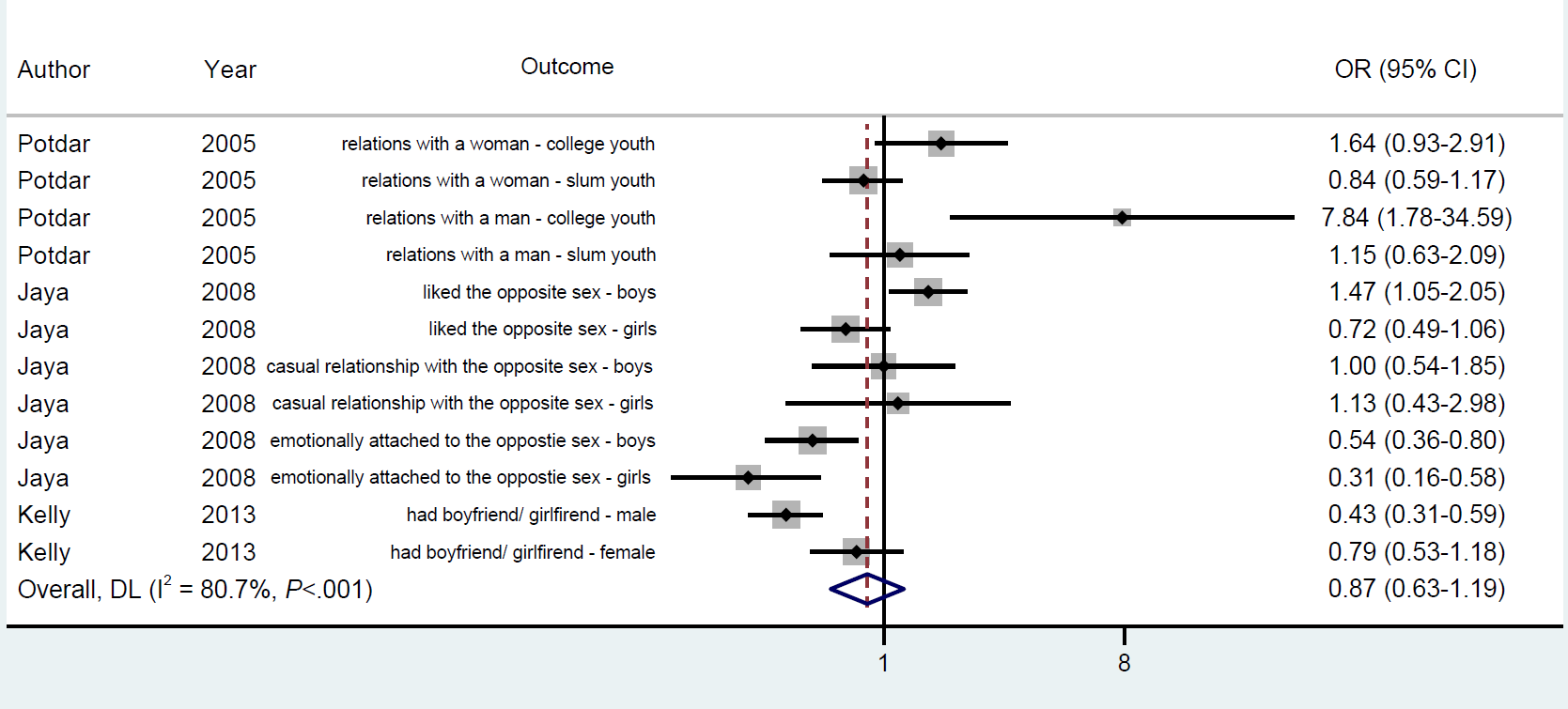


### **Figure S4. Odds ratios (ORs) of reporting “ever had a partner” in the ACASI mode as compared to the FTFI mode. Random-effects model. ACASI: audio computer-assisted self-interview; DL: DerSimonian-Laird effect size variance estimates; FTFI: face-to-face interview.**


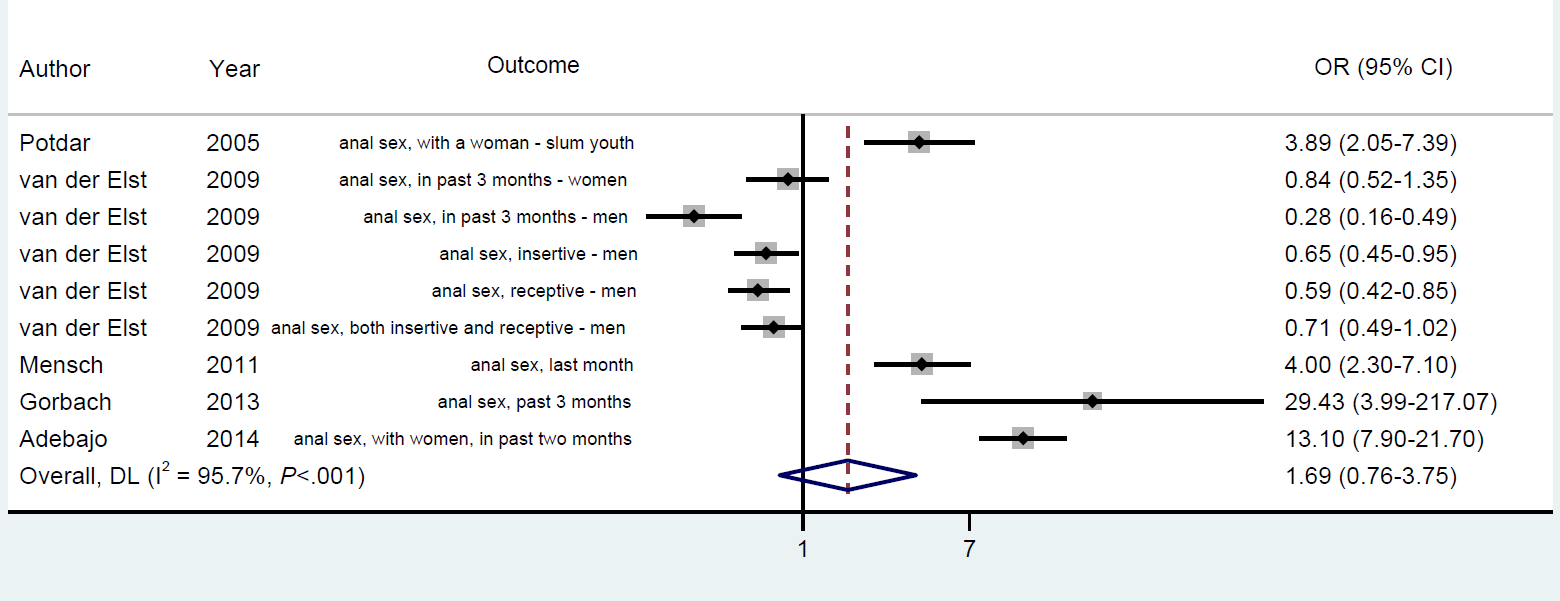


### **Figure S5. Odds ratios (ORs) of reporting “anal sex” in the ACASI mode as compared to the FTFI mode. Random-effects model. ACASI: audio computer-assisted self-interview; DL: DerSimonian-Laird effect size variance estimates; FTFI: face-to-face interview.**
